# Control of human VDAC-2 scaffold dynamics by interfacial tryptophans is position specific

**DOI:** 10.1016/j.bbamem.2016.09.011

**Published:** 2016-12

**Authors:** Svetlana Rajkumar Maurya, Radhakrishnan Mahalakshmi

**Affiliations:** Molecular Biophysics Laboratory, Department of Biological Sciences, Indian Institute of Science Education and Research, Bhopal, India

**Keywords:** Δ*G*^0^, apparent Gibbs free energy of unfolding/folding derived from equilibrium chemical denaturation, Δ*G*^0^_kin_, apparent Gibbs free energy of unfolding/folding obtained from folding/unfolding kinetic (chevron plots), Δ*H*_app_, apparent unfolding cooperativity in thermal denaturation, <τ>,  tryptophan lifetime, CD, circular dichroism, *C*_m_, chemical denaturation mid-point, DDM, dodecyl β-d-maltoside, DiPhPC, diphytanoylphosphatidyl choline, GdnHCl, guanidine hydrochloride, hVDAC-2, human voltage dependent anion channel isoform-2, *K*_SV_, Stern-Volmer quenching constant, *m*, apparent unfolding/folding cooperativity, *ME*_214–216_, average of molar ellipticity values between 214–216 nm, OMM, outer mitochondrial membrane, *R*_g_, radius of gyration, RMSD, root mean square deviation, RMSF, root mean square fluctuation, SAS, solvent accessible surface, *T*_m_, mid-point of thermal denaturation, *T*_m-start_, start temperature of thermal denaturation, WT, wild-type hVDAC-2 protein, VDAC-2, Tryptophans, Stability, Thermodynamics, Kinetics

## Abstract

Membrane proteins employ specific distribution patterns of amino acids in their tertiary structure for adaptation to their unique bilayer environment. The solvent-bilayer interface, in particular, displays the characteristic ‘aromatic belt’ that defines the transmembrane region of the protein, and satisfies the amphipathic interfacial environment. Tryptophan—the key residue of this aromatic belt—is known to influence the folding efficiency and stability of a large number of well-studied α-helical and β-barrel membrane proteins. Here, we have used functional and biophysical techniques coupled with simulations, to decipher the contribution of strategically placed four intrinsic tryptophans of the human outer mitochondrial membrane protein, voltage-dependent anion channel isoform-2 (VDAC-2). We show that tryptophans help in maintaining the structural and functional integrity of folded hVDAC-2 barrel in micellar environments. The voltage gating characteristics of hVDAC-2 are affected upon mutation of tryptophans at positions 75, 86 and 221. We observe that Trp-160 and Trp-221 play a crucial role in the folding pathway of the barrel, and once folded, Trp-221 helps stabilize the folded protein in concert with Trp-75 and Trp-160. We further demonstrate that substituting Trp-86 with phenylalanine leads to the formation of stable barrel. We find that the region comprising strand β4 (Trp-86) and β10-14 (Trp-160 and Trp-221) display slower and faster folding kinetics, respectively, providing insight into a possible directional folding of hVDAC-2 from the C-terminus to N-terminus. Our results show that residue selection in a protein during evolution is a balancing compromise between optimum stability, function, and regulating protein turnover inside the cell.

## Highlights

• Aromatic belt of membrane proteins has key stabilization role.
• Human voltage-dependent anion channel isoform-2 (hVDAC-2) has four interfacial indoles.
• Tryptophans act in concert to drive folding and stabilization of the barrel.
• The 86th position shows preference for phenylalanine due to its buried environment.
• Strands β10–14 promote barrel folding and stabilize hVDAC-2.

## Graphical abstract

**Figure:**
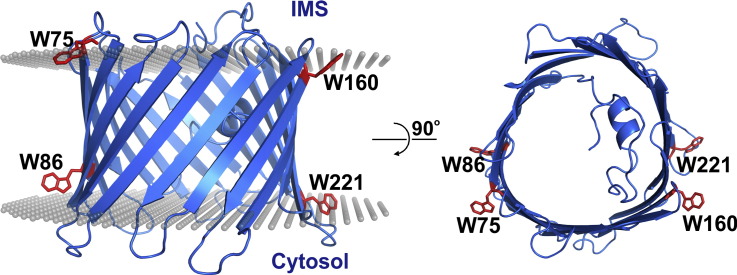

## 1 Introduction

Integral membrane proteins require appropriate positioning of amino acids that help in anchoring the folded scaffold to the amphipathic interface of the bilayer membrane. They require hydrophilic residues in the loops and solvent-exposed extramembrane segments to interact with the aqueous environment and lipid head groups, while hydrophobic residues accommodate themselves towards the tail region of lipids. Tryptophan, tyrosine and lysine are usually found in the region at the interface of the lipid molecules and aqueous environment. These residues help in docking the transmembrane segment of the protein within the membrane due to their ability of ‘snorkeling’ at the interface [1], [2]. Tryptophans and tyrosines often form an ‘aromatic belt’ in membrane proteins that stabilize the native structure of the protein, and contribute immensely to the overall protein stability [2], [3], [4]. Considering the high cost of tryptophan synthesis in the cell, particularly in lower organisms [5], the indole moiety is incorporated judiciously in proteins. Hence, the enrichment of tryptophans in membrane proteins [6] illustrates the importance of this aromatic residue for the overall protein scaffold formation and its stability within membranes.

Tryptophan is not only the most polarizable residue, but also has the capability to form a repertoire of interactions including hydrogen bonds, π-stacking, hydrophobic interactions, N—H...π, and C—H...π [7]. The diverse interaction network involving tryptophan allows this residue to serve as a strong anchoring point for membrane proteins. Recent experimental evidence points to a contextual contribution of the indole ring to the overall membrane protein stability [8]. In less polar environments, the contribution of tryptophan is highest at the lipid hydrophobic core, while in environments that resemble cellular conditions, the greatest stabilizing contribution for tryptophan was seen at the interface [8]. An overall stabilizing effect of tryptophans is, however, anticipated, depending upon its positioning according to membrane depth [9]. Furthermore, previous studies on poly-leucine helix forming peptides have suggested that while phenylalanine is energetically stabilized towards the hydrophobic lipid core [10], tryptophan pulls the peptide to the lipid-water interface [1].

Extensive analysis using the transmembrane β-barrel model protein OmpA has revealed that the ‘*driving force for folding and stability*’ is conferred by the interface aromatics [11]. Such stabilizing effects are magnified by the formation of aromatic clusters. This is facilitated by a preferential localization of the phenylalanine side chain towards the lipid core, with positioning of tryptophan and tyrosine towards the interface [8]. Free energy values derived for the aromatic residues using other transmembrane barrels such as OmpX and Ail establish an important role for interface tryptophans to the folding of β-barrels as well as the post-folding structural stabilization [12], [13]. Indeed, in the case of the bacterial outer membrane β-barrel PagP, a solvent-exposed tryptophan located in the N-terminal helix was found to be critical for anchoring the folded barrel to the membrane [14], [15].

The thermodynamic and folding studies of bacterial outer membrane proteins have helped us understand how interfacial tryptophans stabilize transmembrane β-barrel proteins [8], [11], [12], [13]. As mitochondria possess a bacterial ancestry, one may presume that β-barrels of the mitochondrial outer membrane, such as the translocases, sorting and assembly machinery, and porins also possess interfacial tryptophans that confer a stabilizing role to the protein scaffold. However, experimental evidence in this direction is still lacking. In this study, we address the importance of interfacial tryptophans of the human voltage-dependent anion channel isoform 2 (hVDAC-2). hVDAC-2 is one of the porins found in the mitochondrial outer membrane of eutherian mammals, and is involved in maintaining metabolite flux [16]. Using biophysical techniques, we have examined the contribution of tryptophans and consequence of its substitutions on the folding, stability, and function of this outer mitochondrial membrane (OMM) transmembrane β-barrel protein.

**Fig. 1:**
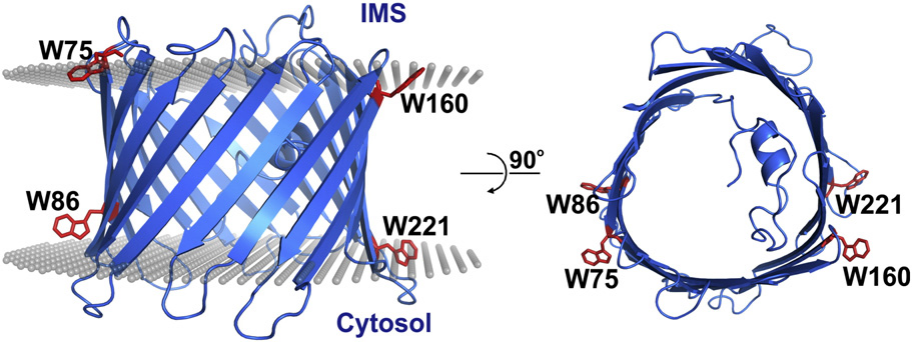
Schematic of I-TASSER modeled hVDAC-2 WT barrel showing the side (left panel) and top view (right panel). The barrel (shown as blue cartoon) highlights the positions of the four tryptophans (red sticks) with respect to the bilayer membrane (grey spheres in the left panel). Of the four intrinsic tryptophans, three (Trp-75, Trp-160 and Trp-221) lie close to the interface, while Trp-86 lies towards the interior. The position of the bilayer membrane was defined by superposing the hVDAC-2 structure on the zebrafish VDAC-2 structure (PDB ID: 4BUM) obtained from the OPM database [27], [28]. IMS, intermembrane space.

hVDAC-2 belongs to the family of primary channel transporters in the OMM. While it is involved in maintenance of cellular homeostasis and transport of metabolites across the OMM [17], hVDAC-2 mainly contributes to cell survival by binding and inhibiting the Bcl-2 family protein BAK [18]. It is a 19-stranded asymmetric barrel with an N-terminal solvent-exposed helix that docks within the barrel, and is important for voltage gating [19]. While we have a tentative mechanism for hVDAC-1 folding [20], experimental evidences for hVDAC-2 folding is still lacking. hVDAC-2 has four tryptophan residues, all of which are strategically placed at the lipid-water interface, on opposite faces of the barrel (Fig. 1). To study the contribution of these tryptophans to barrel stability and function, we constructed mutants with single tryptophans (W86,160,221F, W75,160,221F, W75,86,221F and W75,86,160F) and single tryptophan substitution mutants (W75F, W86F, W160F, W221F) (Table 1).

Membrane protein interfaces are highly sensitive to mutagenesis [11], as they directly affect the correct positioning of the transmembrane segment in lipid membranes. Hence, we substituted tryptophan with phenylalanine, as the latter provides a similar aromatic nature and preferentially interacts with the hydrophobic region of the lipid. Using fluorescence measurements, we show that tryptophan contribution to the hVDAC-2 barrel stability is position dependent, as seen for other bacterial β-barrels [1], [11], [12], [13]. We find that tryptophans at two positions, namely, 86 and 160, when substituted with phenylalanine, increase the stability of folded hVDAC-2 barrel in detergent micelles. The other two positions can accommodate both Trp and Phe, as the barrel stability remains similar in both cases. However, we find that all Trp → Phe substitutions considerably decrease the secondary structure content of hVDAC-2. Further, tryptophans 75, 86 and 221 are important for hVDAC-2 gating. Hence, we hypothesize that evolution has chosen to retain tryptophans at these positions to maintain a balance between hVDAC-2 stability and function within the cell, which are together very important for maintaining cellular homeostasis.

## 2 Materials and methods

### 2.1 Preparation of folded hVDAC-2 WT protein and its mutants

Cloning of human *VDAC-2* wild type gene was carried out using established protocols [21]. Single and multi-tryptophan mutants were generated using site-directed mutagenesis. The mutants are named according to the position wherein the tryptophan is substituted to phenylalanine (Table 1). Single tryptophan mutants are named as W86,160,221F, W75,160,221F, W75,86,221F and W75,86,160F, corresponding to the presence of single-Trp at 75^th^, 86^th^, 160^th^ and 221^st^ positions, respectively. Multi-tryptophan mutants are named as W75F, W86F, W160F, W221F and W75,86F. For example, W75F has all the tryptophans except at the 75^th^ residue (W75 → F). The mutant where all four tryptophans were replaced with phenylalanine was designated W75,86,160,221F (Trp-less mutant). Studies with this mutant were restricted to global secondary structure analysis and simulations.

All the genes were cloned in pET-3a vector. *Escherichia coli* BL21 (DE3) chemically competent cells were transformed with these plasmids, for protein production in the form of inclusion bodies. Isolation of inclusion bodies was carried out using reported protocols [22] with minor modifications (2.75% triton X-100 was used in the wash step) and further purified by anion-exchange chromatography using established methods [21].

Folding of hVDAC-2 WT protein and its mutants was carried out using reported methods [19], in 19.5 mM n-dodecyl-β-d-maltopyranoside (DDM) micelles prepared in Buffer A (50 mM phosphate pH 7.2, 100 mM NaCl) and 10 mM DTT at 4 °C. A final concentration of 25 μM protein was achieved in the folding mix. All the experiments were conducted by 5-fold dilution of this mix to obtain 5 μM protein and 3.9 mM DDM in Buffer A containing 2 mM DTT, unless specified otherwise [19]. The samples were not dialyzed, and hence contained residual amounts of GdnHCl (0.12 M).

### 2.2 Channel conductance measurements

Functional analysis of the single tryptophan mutants were carried out as described previously [19]. Single channel conductance was measured at +10 mV in a planar bilayer membrane formed from diphytanoylphosphatidyl choline (DiPhPC) containing 0.1% cholesterol. A membrane with at least 20–100 channels was used to derive the *G*/*G*_max_ plots for each protein, using a voltage ramp from –60 to +60 mV at 3 mV/s. The voltage gating parameters *n* and *V*_0_ were calculated as described earlier [19].

### 2.3 Measurement of rate of folding of hVDAC-2

The folding rates of all the proteins at low GdnHCl concentrations (< 0.8 M) were measured at 4 °C using tryptophan anisotropy. Parameters reported earlier [19] were followed for all measurements. Briefly, a λ_ex_ of 295 nm and λ_em_ of 340 nm, each with a slit width of 5 nm, were used for anisotropy measurements. The unfolded protein stock (250 μM) in 6 M GdnHCl was rapidly mixed with 10-fold excess of pre-chilled 19.5 mM DDM in Buffer A and 10 mM DTT. The acquisition was started immediately after the mixing. The instrument dead time was ~ 10 s. This experiment suffers from the limitation that each data point is recorded after ~ 15.74 s; hence, the first data point is acquired at ~ 25 s. The data was normalized to obtain folded fraction (*f*_F_) using the following equation.

(1) $$f_{F}=\frac{r-r_{U}}{r_{F}-r_{U}}$$

Here, *r* is the fluorescence anisotropy at a given time, *r*_F_ and *r*_U_ are the fluorescence anisotropy of folded and unfolded proteins, respectively. The single tryptophan mutants gave better fits to a double exponential function, but the rates obtained from independent experiments were not consistent. Hence, all data was fitted to a single exponential function and the rate of folding was derived for all the mutants. Errors are standard deviation from at least three independent experiments.

### 2.4 CD wavelength scans and thermal denaturation measurements

Far-UV wavelength scans were recorded using circular dichroism (CD) spectropolarimetry to obtain the total secondary structure content of the folded tryptophan mutants. To minimize the contribution of noise to the estimations, an average of molar ellipticity values from 214–216 nm (*ME*_214–216_) was calculated. This was used to decipher the β-sheet content, and hence the folding efficacy, of the hVDAC-2 mutants. The folded protein was also subjected to thermal denaturation (T-scan) from 4–95 °C, and the loss in secondary structure was monitored using far-UV CD at 215 nm. The various thermal denaturation parameters *T*_m_, Δ*H*_app_ and *T*_m-start_ were derived from the T-scan experiments as explained previously [19], [23].

### 2.5 Acrylamide quenching and measurement of fluorescence lifetime of tryptophans

All measurements were carried out using established protocols [19] at 25 °C. The effect of acrylamide quenching was studied using steady-state fluorescence [21] and time-correlated single photon counting (TCSPC) [13]. Briefly, samples were incubated with varying concentrations of acrylamide at 25 °C for 5 min, after which, measurements were carried out. Inner filter correction was applied as described previously [21] for steady-state fluorescence measurements. A λ_ex_ of 295 nm and λ_em_ of 340 nm was used for both steady-state fluorescence and TCSPC measurements. The average lifetime (<τ>) of tryptophan was obtained by fitting the TCSPC data to a triple exponential function [21].

### 2.6 Equilibrium (un)folding measurements using tryptophan fluorescence

Equilibrium folding and unfolding experiments were carried out by using GdnHCl as the chemical denaturant, with minor modifications of previous protocols [19]. An excitation wavelength of 280 nm was used to improve the signal-to-noise ratio for the single tryptophan mutants and was monitored using Trp fluorescence intensity. As some of the mutants showed hysteresis, the apparent thermodynamic parameters (free energy, Δ*G*^0^; cooperativity of folding or unfolding, *m* value; mid-point of denaturation, *C*_m_) were derived for both the unfolding (Δ*G*^0^_U_, *m*_U_, *C*_m-U_) and folding (Δ*G*^0^_F_, *m*_F_, *C*_m-F_) pathways, separately.

### 2.7 Kinetics measurements using tryptophan fluorescence and chevron plots

The folding arm of the chevron plot was generated by manually mixing 25 μM protein in 19.5 mM DDM, 4 M GdnHCl, 10 mM DTT and Buffer A, into a five-fold excess of varying concentrations of GdnHCl (0.8–1.68 M) prepared in Buffer A. For the unfolding arm, 25 μM of the folded protein stock (generated by the folding protocol mentioned in the first section) was diluted 2.5-fold in Buffer A. The obtained mix was then diluted in a 1:1 ratio using a stopped flow accessory (dead time ~ 8 ms) into varying concentrations of GdnHCl (1.72–3 M) prepared in Buffer A. The final reaction mix for creating both the arms contained 5 μM protein in 3.9 mM DDM, 2 mM DTT, and different GdnHCl concentrations in Buffer A. All the measurements were carried out by measuring the change in tryptophan fluorescence intensity, using λ_ex_ = 295 nm and λ_em_ = 340 nm at 25 °C, and the reactions were monitored until no further change in tryptophan fluorescence intensity was observed. For manual mixing, excitation and emission slit widths were 3 nm and 5 nm, respectively, with data accumulation every 6 s to minimize photobleaching. The dead time for manual mixing was ~ 10 s. For stopped flow experiments, excitation and emission slit widths were 2 and 10 nm, respectively, with data acquisition every 0.1 s.

The data was fitted either to a single or double exponential function, and the rates (*k*_f_, rate from folding kinetics; *k*_u_, rates from unfolding kinetics) were plotted against the respective GdnHCl concentrations to obtain the chevron plots. The linear zone of both the arms was fitted to the following equations [24].

(2) $$\ln\left(k_{f}\right)=\ln\left(k_{f}^{H_{2}O}\right)+\frac{m_{F-\mathrm{kin}}\left[D\right]}{\mathrm{RT}}$$

(3) $$\ln\left(k_{u}\right)=\ln\left(k_{u}^{H_{2}O}\right)+\frac{m_{U-\mathrm{kin}}\left[D\right]}{\mathrm{RT}}$$

wherein *k*_f_^H_2_O^ and *k*_u_^H_2_O^ are the intercepts and *m*_F-kin_ and *m*_U-kin_ are the slopes of folding and unfolding arm, respectively, *R* is the gas constant in kcal/mol, *T* is temperature in Kelvin and *D* is the denaturant concentration. As the unfolding arm had a non-linear profile, a quadratic Eq. (3) was also used for fitting the unfolding arm wherein *m*_U1-kin_ parameter accounts for the non-linearity [24].

(4) $$\ln\left(k_{u}\right)=\ln\left(k_{u}^{H_{2}O}\right)+\frac{m_{U-\mathrm{kin}}\left[D\right]}{\mathrm{RT}}+m_{U1-\mathrm{kin}}{\left[D\right]}^{2}$$

The free energy of unfolding (Δ*G*^0^_kin_) was calculated using Eq. (5)

(5) $$\Delta G_{\mathrm{kin}}^{0}=-\mathrm{RTln}\left(\frac{k_{f}^{H_{2}O}}{k_{u}^{H_{2}O}}\right).$$

The Tanford β value (*β*_T_) was calculated as reported previously [25].

(6) $$\beta_{T}=\frac{-m_{F-\mathrm{kin}}}{m_{U-\mathrm{kin}}-m_{F-\mathrm{kin}}}.$$

### 2.8 Molecular dynamics simulations

I-TASSER [26]—modeled hVDAC-2 structure was used as the input file for all the simulations. The structure was first oriented in accordance to zebrafish VDAC-2 (PDB ID: 4BUM) template from Orientations of Proteins in Membranes (OPM) database [27], [28], using PyMOL v1.5.0.5 [29]. The assembled hVDAC-2—micelle structure for simulations was generated using the Micelle Builder tool in the CHARMM-GUI web server [30], [31]. The W75,86,160,221F (Trp-less) mutant was generated by mutating all the tryptophan residues to phenylalanine, using the PDB manipulation options in the input generator. The hVDAC-2 barrel was inserted into a DDM micelle formed from 80 molecules. Increasing the number of DDM molecules did not change the barrel properties. Hence, for further analyses, only 80 DDM molecules were used. The system was hydrated using TIP3P water model and 0.1 M NaCl was added to neutralize the charges, and make the system similar to experimental conditions. The final protein-detergent complex was ~ 110.4 × 110.4 × 110.4 Å^3^ and had 1 protein, 80 DDM molecules, 67 Na^+^, 68 Cl^–^ ions and 38,499–39,095 water molecules.

Initially, three independent short simulations were carried out for ~ 10 ns for both WT and W75,86,160,221F, with different starting protein-detergent complexes. Here, all simulations were carried out at a constant temperature of 298.15 K and 1 bar pressure, using GROMACS v5.0.4 [32]. The system was first energy minimized, and then equilibrated in six steps with the conformational restraint on the system decreasing with each step [31]. A single 100 ns production run was carried out with zero restraints using one of the starting protein-detergent assemblies, on GROMACS v5.0.2, for WT and W0, and used for the final trajectory generation and analysis. Particle mesh Ewald method was used for long-range electrostatic interactions [33] and LINCS algorithm for constraining covalent bonds involving hydrogen atoms [34]. All final analyses were carried out using GROMACS v5.0.4, VMD v1.9.2 [35] or PyMOL. A sausage representation was generated from the average structure using B-factors in PyMOL.

## 3 Results

### 3.1 Single tryptophan mutants show altered voltage dependence

The VDAC-2 barrel shows conformational heterogeneity [36] that manifests upon its channel function in the form of diverse conductance states [19], [37]. The observed conductance values at +10 mV are centered at ~ 4 nS and ~ 2 nS and correspond to the open and subconductance channel states [19]. The influence of hVDAC-2 cysteines on channel behavior is documented [19]. However, the role of tryptophans in VDAC-2 channel functioning is not known. Hence, we probed the effect of tryptophan mutation on hVDAC-2 channel activity on a planar lipid bilayer system. As all the four tryptophans are positioned facing the lipid membrane, a drastic change in hVDAC-2 function was not expected upon conserved substitution with phenylalanine.

**Fig. 2:**
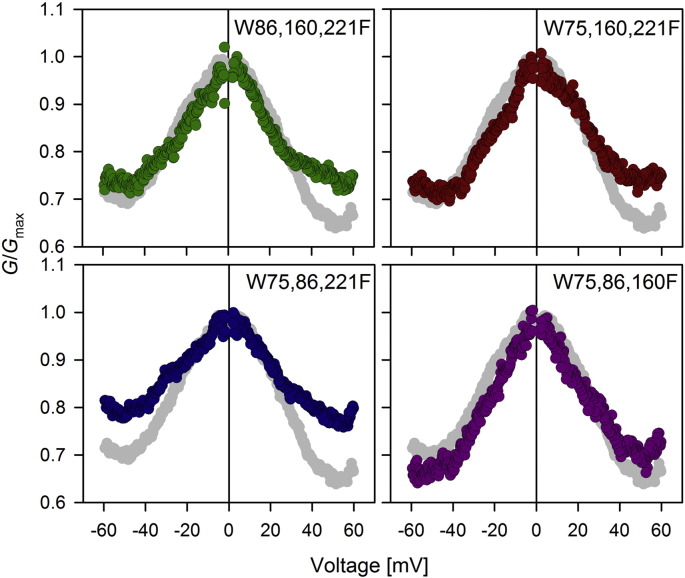
*G*/*G*_max_ plots for all the four single tryptophan mutants derived from multi-channel membranes. Both W86,160,221F and W75,160,221F show minor variation in the subconductance states at positive voltages. W75,86,221F, on the other hand shows considerable variation at both the positive and negative voltages, and is least sensitive to increasing voltage. Grey symbols indicate the WT protein and is shown here for purpose of comparison. Error bars are omitted for the sake of clarity. The complete data is shown in Supplementary Fig. 1. Data for hVDAC-2 WT protein has been reproduced from reference [19] with permission.

All the four single tryptophan mutants insert at varying conductance levels at +10 mV (Table 2), as observed previously for the hVDAC-2 WT protein [19]. This shows that mutation of tryptophans does not alter the conductance states seen upon channel insertion, to a considerable extent. When a multichannel membrane containing these mutants is subjected to triangular voltage ramps ranging from –60 mV to +60 mV, we observe marginally steeper voltage dependence for almost all single tryptophan mutants, when compared to the WT (Table 2). hVDAC-2 W86,160,221F shows an additional decrease in the *V*_0_ values at positive voltages (Table 2 and Supplementary Fig. 1). Surprisingly, W75,86,221F shows a subconductance state that is slightly different from other tryptophan mutants (lower left panel of Fig. 2). The tryptophan to phenylalanine substitutions carried out in this study are not expected to drastically alter VDAC-2 functional characteristics when compared with other mutations, which change the charged state or chemical nature of the substituted amino acid [38], [39], [40]. Hence, the subtle functional differences we observe in a background of conserved substitutions might be sufficient for interpretation. Our data signifies the importance of tryptophans at 75, 86 and 221 in channel gating. The subconductance state achieved at higher voltages displays higher conductivity values when compared to WT, as the response to the applied voltage for this mutant barrel is weak.

We calculated the *nFV*_0_ value, which is an indication of the difference in the energy of open and closed states (Table 2) [41]. All the mutants show a decrease in *nFV*_0_ value, demonstrating a certain degree of conformation rearrangement upon replacement of tryptophans [41]. We find that although tryptophans may not be important for maintaining the pore diameter as they face the lipids, they do affect the overall energy difference between the open and the closed states of the lipid-inserted barrel.

### 3.2 Folding proceeds from N- to C-terminus in hVDAC-2 barrel formation

Membrane protein folding and factors affecting this process have previously been studied in lipidic micelles and vesicles. We have previously shown that hVDAC-2 WT exhibits rapid folding rates in detergent micelles, while folding in vesicles is inefficient [21]. Hence, we investigated the effect of tryptophan substitutions on the kinetics of hVDAC-2 barrel folding in dodecyl β-d-maltoside (DDM). Our previous experiments indicate that the overall barrel scaffold is well supported by the oblate DDM micelles [19].

**Fig. 3:**
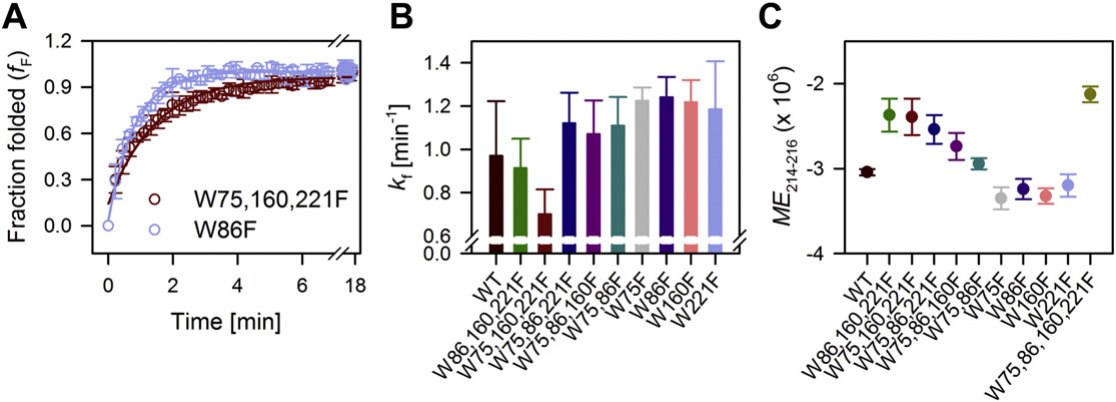
Folding rates and secondary structure content of hVDAC-2 WT and various tryptophan mutants. (A) Folding kinetics of hVDAC-2 W75,160,221F and W86F monitored using tryptophan fluorescence anisotropy in 19.5 mM DDM at 4 °C, highlights the difference in the rates between the two mutants. Solid lines indicate fits to a single exponential function. The folding kinetics for all the mutants and the raw anisotropy data is shown in Supplementary Fig. 2. (B) Histograms summarizing the rates obtained from (A), after fitting individual data to a single exponential function. hVDAC-2 W75,160,221F shows the slowest folding rate in DDM micelles. The initial dead time of the experiment was ~ 25 s, and the initial folding phase could not be accurately captured. Error bars specify standard deviation from three independent experiments. (C) *ME*_214–216_ shows an average of molar ellipticity (ME) values from 214–216 nm, and is used as an indicator of the secondary structure content in folded hVDAC-2. W75,86,160,221F (Trp-less) and all the single tryptophan mutants show a compromised folding efficiency in DDM micelles, and therefore, have lower ME values. Put together, these data highlight the importance of tryptophans for proper folding of the hVDAC-2 barrel.

We used tryptophan fluorescence anisotropy to monitor the folding kinetics at 4 °C, as tryptophan fluorescence intensity measurements are too fast to be captured accurately at GdnHCl concentrations < 0.8 M [21]. As the protein folds, the fluorescence anisotropy of tryptophan also increases. This is because the indole ring undergoes a change from a solvent-exposed flexible conformation in the unfolded protein to a buried and rigid state in the folded protein. The W75,160,221F mutant, which only possesses Trp-86, shows the slowest folding rate among all the mutants (~ 30% slower than WT) (Fig. 3 and Supplementary Fig. 2). This tryptophan lies in β4 strand. Similarly, the W86,160,221F mutant possessing only Trp-75 also folds slowly. This tryptophan lies in the neighboring β3 strand. Mutants with a single Trp at either the 160^th^ (W75,86,221F) or 221^st^ (W75,86,160F) position fold marginally faster than WT hVDAC-2 (Fig. 3). These tryptophan residues are found in strands β10 and β14, respectively.

The folding of hVDAC-2 is a multi-step process [19]. We are monitoring the folding process of hVDAC-2 using the change in Trp fluorescence as a reporter of the barrel folding rate. Hence, our fluorescence measurements can possibly capture the process of hVDAC-2 folding and assembly. If hVDAC-2 follows a directional assembly, the measured rate of change in fluorescence would indicate whether a specific indole is buried early during the folding process. Accordingly, we find that Trp-160 (W75,86,221F) or Trp-221 (W75,86,160F) show faster folding rates. Therefore, one can assume that strands β10–β14 assemble early during hVDAC-2 folding. Similarly, Trp-75 (W86,160,221F) and Trp-86 (W75,160,221F) show slower folding rates than the WT, suggesting that strands β3–β4 assemble later during folding. The folding rate of the WT protein is an average of the folding rates obtained for the single-Trp mutants. Hence, one possible explanation for the data is that the barrel folding proceeds from the C-terminus (where Trp-160 and Trp-221 reside; region corresponding to β10–β14) to the N-terminus (where Trp-86 resides; region corresponding to β4).

It can also be argued that the W → F substitutions at the 160^th^ and 221^st^ positions adversely affect the folding rate of W75,160,221F and to some extent, W86,160,221F. Both of these mutants do not have Trp-160 and Trp-221. To examine this further, we measured the folding rate of the multi-Trp mutants (Fig. 3B). We find that all the mutants that possess both Trp-160 (present in β10) and Trp-221 (present in β14), namely W75,86F, W75F, and W86F display folding rates comparable to (or better than) the WT barrel. Furthermore, the multi-tryptophan mutants W160F and W221F also show similar rates as the WT protein (Fig. 3B). Put together, the second possible explanation for our data is that the presence of either Trp-160 or Trp-221 is sufficient to drive proper barrel folding.

As tryptophans play an important role in the barrel folding and stability, we probed the overall final folded state of the mutant barrels by measuring their far-UV CD profiles (*ME*_214–216_, ellipticity values at 214–216 nm are plotted in Fig. 3C). The secondary structure content of all the single Trp mutants is lower than the WT protein, with W86,160,221F and W75,160,221F remaining the least structured of the mutants. Substitution of all tryptophans with phenylalanine affected barrel folding the most (W0 mutant). The *ME*_214–216_ values, which represent the β-sheet content, follow the order WT ≥ Trp-221 ≥ Trp-160 ≥ Trp-86 ≥ Trp-75 ≥ Trp-less mutant (see Fig. 3C).

When we put together the results from the folding rates and secondary structure analysis, we can conclude that either Trp-160 or Trp-221 is likely to facilitate the intrinsic fast folding of hVDAC-2. Therefore, Trp-160 and Trp-221 are likely to be a part of the folding nucleus and substitution of both these residues with phenylalanine drastically affects the rate of folding and the final folded state of hVDAC-2. The secondary structure content we obtain from the CD measurements supports our assumption from folding rates (Fig. 3B), that folding proceeds from β14 to β4 in hVDAC-2. Further, the multi-tryptophan mutant data indicate that the presence of either Trp-160 or Trp-221 is important for maintaining the well-folded state of the barrel. Such a mechanism of folding, involving nucleation of folding at the C-terminal strands and completion of folding towards the N-terminal strands has also been observed for the 22-stranded β-barrel FhuA [42].

### 3.3 Lone indoles of single-tryptophan hVDAC-2 mutants are buried to a greater extent than WT

We next probed the tryptophan environment using acrylamide accessibility monitored using both steady state tryptophan fluorescence and lifetime (Supplementary Fig. 3). Higher values of the Stern-Volmer constant (*K*_SV_) is obtained in acrylamide quenching measurements if tryptophan is accessible to the quencher (acrylamide). On the contrary lower values of fluorescence liftetime (<τ>) are generally obtained for the solvent-exposed tryptophan residues. All the multi-tryptophan mutants (WT, W75,86F, W75F, W86F, W160F, and W221F) show similar values for the Stern-Volmer constant (*K*_SV_) (Supplementary Fig. 3A). Further, the lifetimes decrease upon acrylamide addition (Supplementary Fig. 3B). The single tryptophan mutants show different lifetimes (between 2–3 ns), lowered *K*_SV_ values (indicating that the indoles have less accessibility to acrylamide as compared to the WT barrel), and different fluorescence quenching profiles (Supplementary Fig. 3). When single tryptophan mutants of hVDAC-2 are probed using acrylamide quenching coupled lifetime measurements, we see that mutant with Trp-75 reaches saturation faster than the other mutants (Supplementary Fig. 3B). This indicates a likely added contribution of a vicinal quencher. The neighboring residue of Trp-75 is Cys-76 (see Fig. 7D), which can act as a potent quencher for tryptophan fluorescence [43].

When three tryptophans are replaced with the more hydrophobic phenylalanine in the WT barrel, we conjecture that the benzyl ring preferentially buries itself, leading to a marginally altered barrel structure. This change, in turn, renders the lone tryptophan inaccessible to acrylamide. Such a behavior for phenylalanine has been reported previously in transmembrane helices [1]. Therefore, a lone tryptophan, particularly at 75^th^ or 86^th^ position, is insufficient to define the barrel interface of hVDAC-2.

### 3.4 Trp-86 is a key deterrent while Trp-221 is a key determinant of hVDAC-2 barrel stability

The four intrinsic tryptophans of hVDAC-2 are placed strategically on the opposing faces (Fig. 1) of the barrel, to define the barrel interface correctly. To better understand the role of each of these tryptophans in barrel stability we subjected the mutants to equilibrium folding and unfolding measurements in DDM micelles, using GdnHCl as the denaturant. The hVDAC-2 WT barrel shows complete reversibility in its (un/re)folding process (Supplementary Fig. 4A). W160F shows considerable hysteresis under these conditions. This mutant retains the three other tryptophans of hVDAC-2, highlighting that the presence of tryptophan at the 160th position is important to maintain the thermodynamic equilibrium of hVDAC-2.

**Fig. 4:**
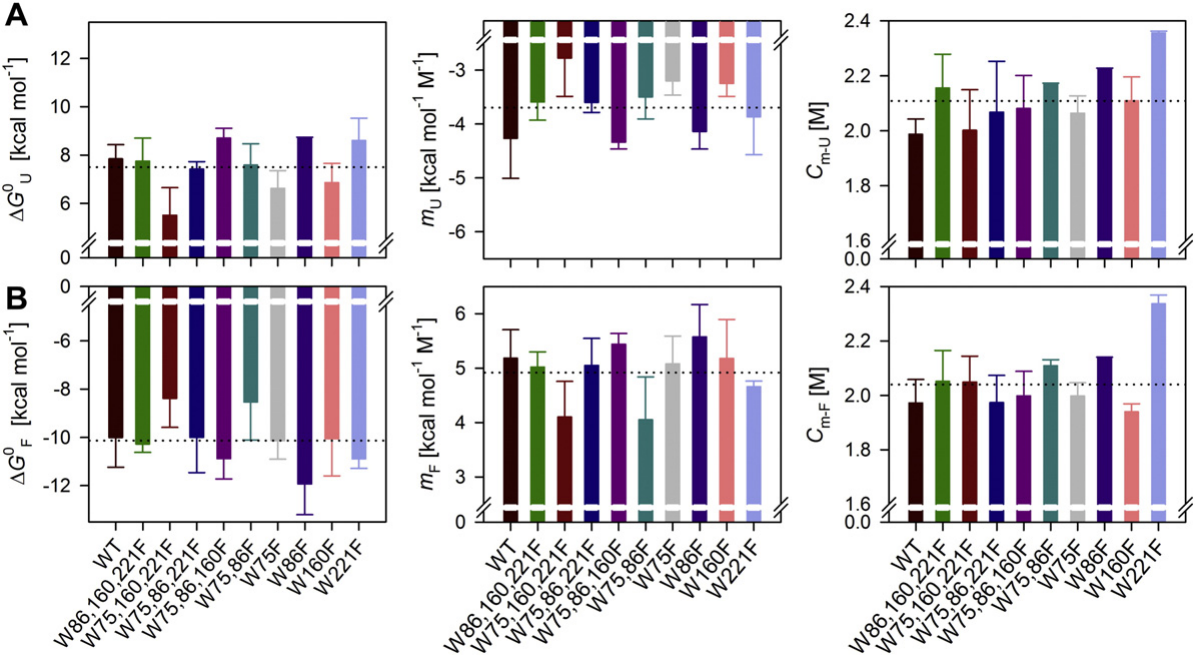
Thermodynamic parameters for hVDAC-2 WT and its various tryptophan mutants. Data was obtained from equilibrium (un)folding measurements in 3.9 mM DDM after a 24 h incubation in a gradient of GdnHCl, at 25 °C. A change in the tryptophan fluorescence intensity was used to monitor the progress of the reaction over 48 h. The equilibrium measurements showed hysteresis (see Supplementary Fig. 4 for more details) for most of the mutants, and hence the thermodynamic parameters derived from unfolding (A) and folding (B) measurements have been shown separately. Among the single tryptophan mutants, W75,160,221F mutant is highly destabilized in both unfolding and folding measurements (lowest Δ*G*^0^ and *m* value), while its counterpart W86F is among the most stable mutants. In general, free energy for folding is higher as compared to unfolding for all the mutants. This is due to the increase in the cooperativity of the folding process (higher *m*_F_), while the *C*_m_ remains largely similar. Dotted lines in each graph indicate the average value for the measured parameter. Error bars denote standard deviations obtained from at least two independent experiments.

We observe hysteresis to different extent in most of the mutants. Further, increasing the incubation time for more than 48 h, led to protein aggregation at intermediate GdnHCl concentrations, especially in the folding reactions (Supplementary Fig. 4B). Hence, we derived apparent thermodynamic parameters [44], for both the unfolding and folding pathways from the 24 h data. This includes the Gibbs free energy (Δ*G*^0^), cooperativity of the unfolding or folding process (*m* value), and mid-point of chemical denaturation (*C*_m_). In general, we observe that for all mutants, the folding pathway is more cooperative (higher *m* value) and correspondingly, has a higher Δ*G*^0^ than the unfolding pathway (compare Fig. 4A and B). This shows that folding of the hVDAC-2 barrel is energetically favorable and under optimal conditions, the equilibrium is shifted towards the folded state. The unfolding process, on the other hand, is less cooperative, which can be due to slower denaturant access to the folded barrel upon GdnHCl addition. We also see protein aggregation in all the mutants at lower GdnHCl concentrations, upon prolonged incubation, as observed earlier in the WT barrel [19]. Hence, the aggregation process at lower GdnHCl concentrations is independent of tryptophan residues.

We see a drastic decrease in protein stability when only tryptophan at the 86^th^ position is present (W75,160,221F, Fig. 4), in both the unfolding and folding pathways. Hence, tryptophans at positions 75, 160 and 221 are important for hVDAC-2 stability. The low free energy for W75,160,221F is mainly due to low *m* values, as the *C*_m_ is similar to the WT for all the single Trp mutants. According to the modeled structure, the 86^th^ position is comparatively more buried than the other three tryptophans, which are at the interface (Fig. 1). We find that while the other positions can accommodate both tryptophan and the more hydrophobic phenylalanine without considerably changing barrel stability, phenylalanine at 86^th^ position increases stability. This is also confirmed by the ~ 1.7 fold increase in stability of the W86F mutant over the W75,160,221F mutant, wherein, W86F differs from the WT barrel at just the 86^th^ position (Fig. 4). The W75,86F mutant is also destabilized to a similar extent as the W75,160,221F mutant in the folding process, due to lowered *m* value. We conclude that a highly cooperative folding process of hVDAC-2 is achieved if at least two tryptophans (Trp-75 and Trp-160/Trp-221) are present at opposite faces (β3 and β10/β14) of the barrel.

Residue 221 displays interesting features in our experiments. First, W75,86,160F is the only single-Trp mutant to display cooperative protein folding and unfolding, comparable to the WT protein. Secondly, the mutation of Trp-221 (in the W221F mutant) shows a marginally lowered *m* than the other multi-Trp mutants (Fig. 4). Hence, the 221^st^ Trp contributes to the folding and stability of hVDAC-2. Interestingly, when the 221^st^ position has phenylalanine (W221F mutant), the barrel exhibits increased resistance to chemical solvation (highest *C*_m_ value) (Fig. 4). Indeed, this is the only construct to exhibit a considerably different *C*_m_. Residue 221 is present near to one of the longest loops of the hVDAC-2 protein. A mutation in this region can have implications on the strand registry, thereby influencing the chemical solvation of the barrel. We speculate that barrel folding proceeds from the C- to N-terminus (β14 and β10 fold before β4; see Fig. 1). We can explain the ability of Phe-221 to nucleate folding through initial interaction with detergent molecules when we consider the hydrophobic nature of phenylalanine. This property can drive folding of the W221F barrel at higher GdnHCl concentrations, and account for its high *C*_m_.

Overall, a global reduction in the unfolding free energy across all mutants is due to the lowered unfolding cooperativity (low *m* values). The destabilizing effect of Trp at the 86^th^ position and favorable contribution from 221^st^ position in hVDAC-2 folding are also conserved in the unfolding pathway.

### 3.5 β3-4 is inherently unstable during folding of hVDAC-2 barrel in DDM micelles

Most of the tryptophan mutants showed hysteresis in equilibrium experiments. Hence, we validated our deductions using the thermodynamic parameters obtained from chevron plots. It must be noted here that on account of hysteresis, the kinetic traces saturate to an intermediate state for the GdnHCl concentrations lying in the transition zone of our equilibrium experiments (~ 1.5 M–2.5 M). We obtained the folding arm by manual mixing, and by monitoring the change in the fluorescence intensity of tryptophan as the barrel folds. The folding kinetics fit well to a double exponential function (Supplementary Figs. 5 and 6). This shows that folding of the hVDAC-2 barrel proceeds through two steps. The initial association of the barrel with the micelle, upon dilution of the denaturant, is likely to be rapid, representing the fast phase. Minor structural rearrangements, especially in the vicinity of the 86^th^ position decide the slow phase. We also observed a marginal rollover in the folding arm at very low GdnHCl concentrations, indicating the likely presence of an intermediate. Additionally, we also knew from our equilibrium measurements that hVDAC-2 barrel has a tendency to aggregate at low GdnHCl concentrations, which can also cause this rollover [25]. Hence, we used only the linear region of the folding arm for our subsequent analysis.

The unfolding arm, on the other hand, is very shallow and had a smooth rollover at ~ 2.5 M GdnHCl (Supplementary Figs. 5 and 6). Here, the tryptophan fluorescence data obtained at low GdnHCl concentration could be defined well with a single exponential function. As the GdnHCl concentration increases beyond ~ 2.3 M, the kinetics fit well to double exponential function. We fit the unfolding arm both to a linear function (Supplementary Fig. 5), and with a function that considered the rollover (Supplementary Fig. 6), to derive the thermodynamic parameters. As anticipated, fits to the linear function gave us free energy values (Δ*G*^0^_kin_) that were marginally lower than the latter (Supplementary Table 1); however, the trend in free energy differences among the mutants was consistent with both methods of data analysis. Overall, the chevron analysis indicates the presence of intermediates in hVDAC-2 folding, and the Δ*G*^0^_kin_ does not account for the total change in the free energy of the system. Hence, the kinetic free energy is underestimated for hVDAC-2 barrel in DDM micelles in our experiments.

Upon comparing the thermodynamic parameters obtained from chevron analysis, we find that W75,160,221F is the least stable of all mutants, followed closely by the W86,160,221F construct. Our kinetics study highlights the inherent instability of the β3-4 region in the barrel. We derived the Tanford β value, which represents how compact the intermediate state is with reference to the folded and unfolded protein states. The Tanford β values are ~ 0.5 for all the mutants, indicating that the transition state lies between the native and denatured states [45], [46], and is not affected by substitution of tryptophan. Considering all these results, we find that unfolding of WT takes place through an intermediate that is not detected in the equilibrium chemical denaturation experiments. Additional experiments that allow for the capture of the unfolding intermediate are needed to characterize its nature. Further, hVDAC-2 W75,160,221F and W86,160,221F have compromised folding kinetics presumably due to the structural rearrangement of the β3–4 segment being a slow process and/ or the W → F replacement at residues 160 and 221.

### 3.6 Positions 75, 160 and 221 act in concert to determine hVDAC-2 global thermal stability

**Fig. 5:**
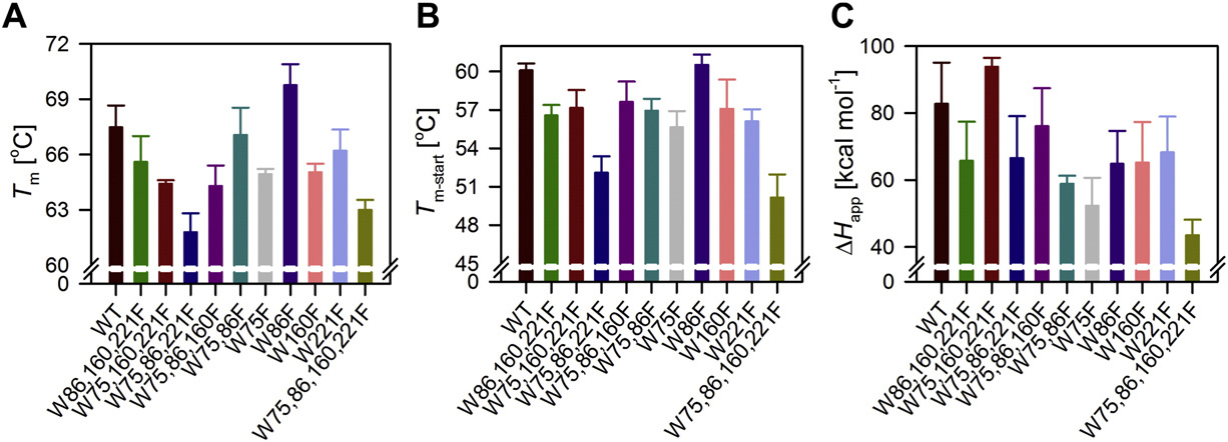
Summary of parameters derived from thermal denaturation experiments of hVDAC-2. Denaturation of the barrel structure was monitored using far-UV CD in 3.9 mM DDM. *T*_m_, midpoint of thermal denaturation (A), *T*_m-start_, starting temperature of secondary structure collapse upon heating (B), and Δ*H*_app_, cooperativity of unfolding (C) are derived from the T-scan experiments shown in Supplementary Fig. 7B. hVDAC-2 W75,86,221F and W75,86,160,221F mutants are among the least thermal stable mutants, while W75,86,221F exhibits the highest cooperativity of unfolding. Note, however, that W86F possesses the highest *T*_m_.

We addressed the effect of interfacial hVDAC-2 tryptophans on the global folded structure and stability of the folded barrel in DDM micelles. For this, we subjected the folded protein to thermal denaturation from 4–95 °C and monitored the change using far-UV CD at 215 nm. hVDAC-2 undergoes aggregation upon unfolding [19]. The far-UV CD data provides information for both unfolding and aggregation, which we can obtain from the *T*_m_ and *T*_m-start_ (mid-point and unfolding start temperatures, respectively), and the apparent change in the overall enthalpy (Δ*H*_app_), which is also an indication of the overall cooperativity of the reaction [19]. The Trp-less mutant (W75,86,160,221F) is one of the most unstable mutants, with lowest *T*_m_ and *T*_m-start_ (mid-point and unfolding start temperatures, respectively) values. The Δ*H*_app_ is also lowest for Trp-less mutant (Fig. 5). Hence, tryptophans play an important role in anchoring the folded barrel to the micelles, and its removal causes the barrel to unfold in a less cooperative manner over a large temperature range (Supplementary Fig. 7).

All single and multi-tryptophan mutants show lowered *T*_m_ (Fig. 5A). In line with our findings from chemical denaturation, hVDAC-2 W75,160,221F appears destabilized and shows rapid (cooperative) unfolding (high Δ*H*_app_; Fig. 5C). We believe that due to the initial destabilization and vulnerable folded state of the barrel, hVDAC-2 W75,160,221F collapses rapidly upon heating. On the other hand, W86F mutant emerges as most resistant to thermal denaturation (highest *T*_m_; Fig. 5A). Here again, we find that stability increases when Trp at the 86^th^ position is replaced with a phenylalanine, owing to the buried nature of this region (see Fig. 1). The folded state of W86,160,221F is also comparable to W75,160,221F (Fig. 3C); however, it is not as destabilized (see stability measurements in Fig. 4), and hence unfolds slowly, showing a lower Δ*H*_app_ (Fig. 5C).

**Fig. 6:**
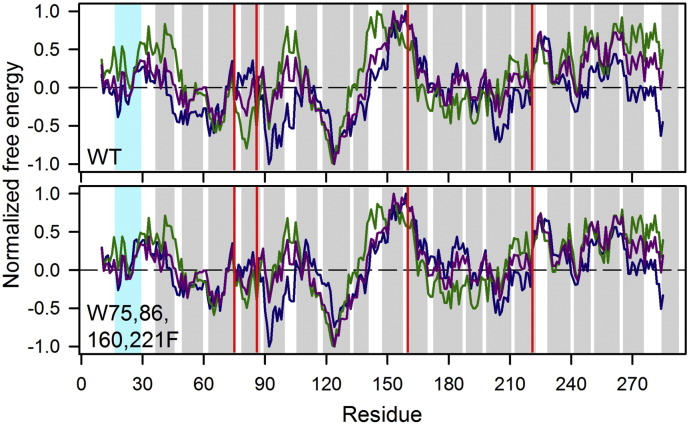
Normalized hydropathy plots for hVDAC-2 WT and W75,86,160,221F (Trp-less) proteins. Hydropathy plots were made using the Wimley-White interfacial scale (dark blue, [47]), Kyte & Doolittle plot (dark green, [48]) and transmembrane tendency scale (purple, [49]) with a window size of 9. Regions corresponding to the β-strands are shaded in grey, and the N-terminal helix (residues 17–29) is indicated as cyan. Solid red drop lines mark positions 75, 86, 160 and 221. Residues in the vicinity of position 160 show the highest partition energy, and also lie in a hydrophobic zone.

An unexpected finding from the thermal denaturation experiments came from the W75,86,221F mutant. This mutant shows stability levels (lowest *T*_m_) comparable to the Trp-less mutant. When the 160^th^ position is substituted for phenylalanine (W160F mutant), thermal stability is regained only to some extent. To better understand the environment of 160th position, we calculated the hydropathy values for hVDAC-2 WT barrel. Hydropathy values derived using three scales (Wimley-White interfacial scale [47], Kyte & Doolittle scale [48], and transmembrane tendency scale [49]) show that Trp-160 has the highest partition energy, and lies in a zone populated with hydrophobic residues (Fig. 6). Trp-221 also lies in a marginally hydrophobic area, whereas the environments of Trp-75 and Trp-86 are amphipathic.

What we find from our thermal denaturation measurements is that except for residue 86, the positions 75, 160 and 221 have incremental contributions to hVDAC-2 stability. This occurs because none of the permutants can completely recover the *T*_m_ and Δ*H*_app_ of WT. Of the single-Trp mutants, W75,86,221F (mutant retaining Trp-160) is insufficient to thermally stabilize hVDAC-2, whereas barrel destabilization is considerably offset by the presence of Trp-75. The barrel scaffold can accommodate Phe well at position 160, but not to the extent of the 86^th^ position. Hence, we conclude that positions 75, 160 and 221 contribute in an additive manner to stabilize the folded barrel, with a clear preference for Phe only at position 86.

### 3.7 Unique environment of each hVDAC-2 tryptophans allow for different contributions

**Fig. 7:**
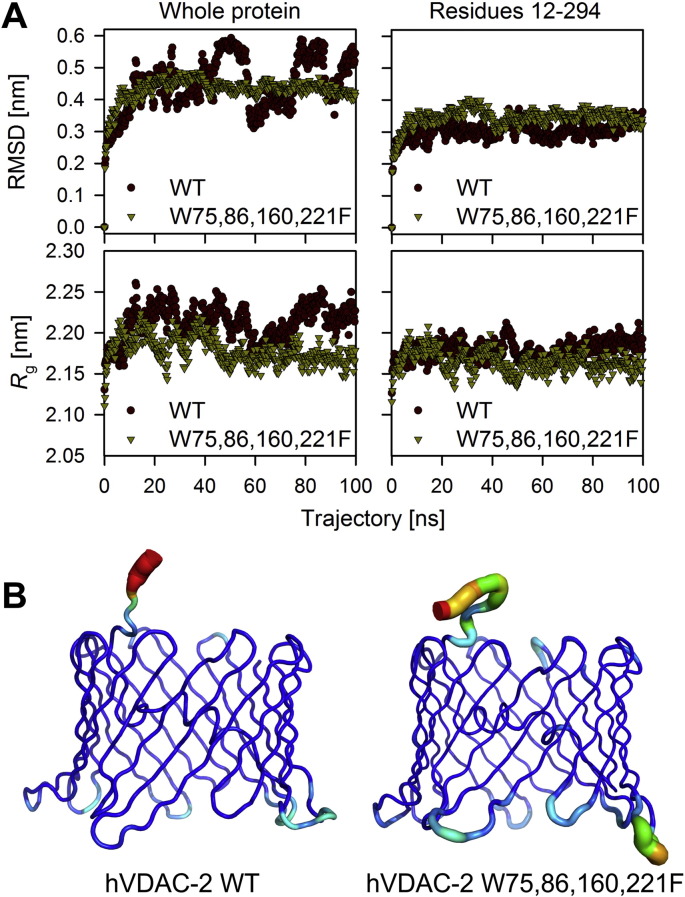
*In silico* analysis of the tryptophan environment in hVDAC-2 barrel probed using molecular dynamics simulations. (A) Comparison of the hVDAC-2 WT and W75,86,160,221F (Trp-less) barrels in DDM micelles along a 100 ns trajectory generated using the GROMACS simulation package [32] using root mean square deviation (RMSD; top) and radius of gyration (*R*_g_; bottom). hVDAC-2 WT barrel shows variation in RMSD and *R*_g_ throughout the trajectory, which is absent in W75,86,160,221F (left panels). This variation stems from the first 11 residues of the N-terminal helix, which exhibit a highly dynamic nature during the simulation. When these residues are not considered in the analysis (right panels), we see that the RMSD and *R*_g_ are invariant for both WT and Trp-less mutant. This suggests that mutating tryptophan to phenylalanine does not alter the stability or the compactness of the barrel. Also see Supplementary Fig. 8 for more information. (B) Sausage representation of WT and Trp-less barrels, showing the dynamic nature of first 11 residues in the WT protein. The structure is color coded based on the B-factor (highest: red; lowest: blue).

We find, from our experimental data, that each hVDAC-2 indole contributes differentially to the voltage gating, folding pathway and post-folding stability of the 19-stranded asymmetric barrel. To further validate our observations, and gain insight into the hydrophobic environment at each Trp position, we carried out atomistic molecular dynamics simulations of hVDAC-2 WT and Trp-less barrels in DDM micelles. Interestingly, when the first 11 residues are not considered, simulations showed no considerable difference in the root mean square deviation (RMSD) and radius of gyration (*R*_g_) of the two mutants (Fig. 7A). The residues 1–11 of N-terminal helix are highly dynamic only in the WT barrel (Fig. 7B), thereby affecting its whole protein RMSD and *R*_g_ values (Fig. 7A, left panels). We observe three zones of stable RMSD values in the 100 ns trajectory with each of the zones corresponding to different orientations of N-terminal region (Supplementary Fig. 8). Further, *R*_g_ represents compactness of the molecule, and simulations showed that substitution of tryptophans to phenylalanines did not considerably alter the compactness of transmembrane region. The root mean square fluctuation (RMSF) and solvent accessible surface (SAS) area are also similar for both the barrels (Supplementary Fig. 9) showing that the barrel topology remains largely unchanged after the substitution. Hence, our simulation studies indicate that a W → F substitution in hVDAC-2 lowers the dynamicity of the N-terminal helix residues 1–11. The reason for this observation is presently unclear.

**Fig. 8:**
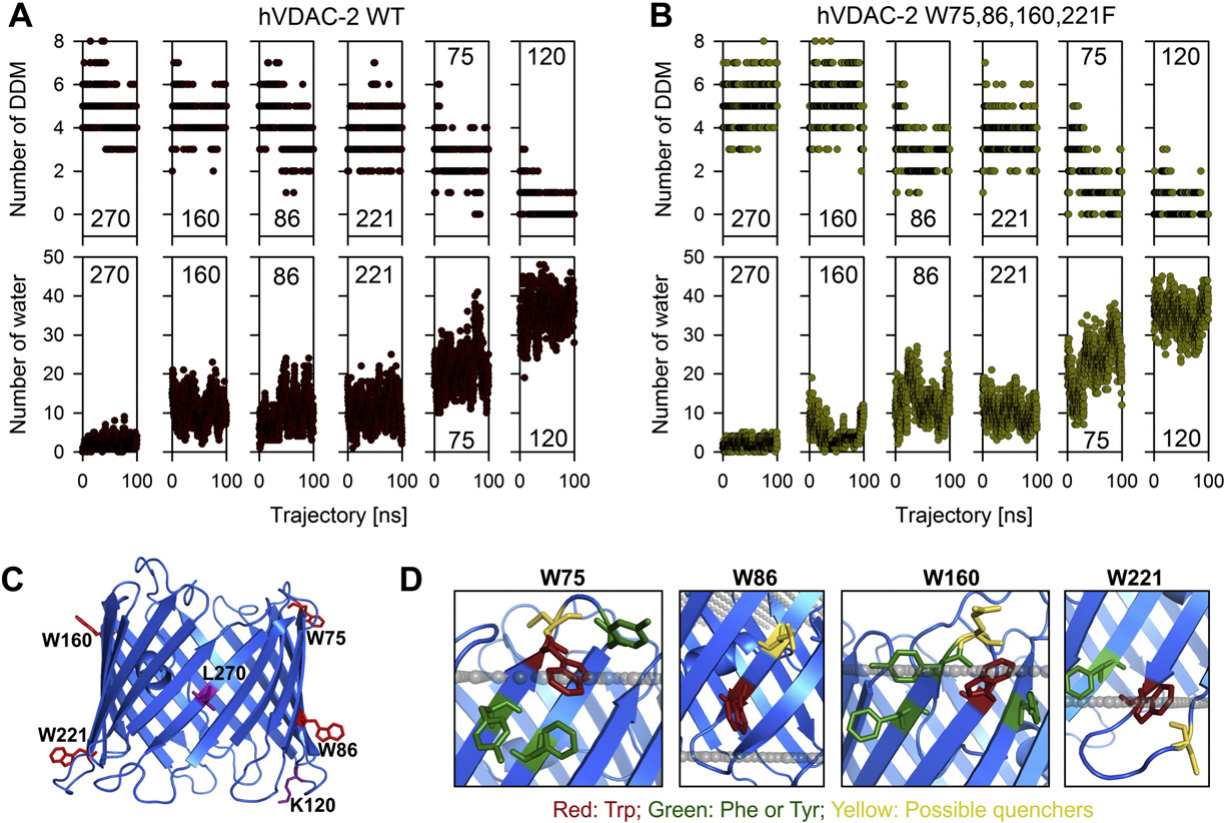
Vicinity analysis of Trp/Phe from simulation studies. Analysis of Trp or Phe at positions 75, 86, 160, 221, and control residues (120 and 270) in a 100 ns simulation of hVDAC-2 WT and W75,86,160,221F in DDM micelles. DDM/water molecules within a 5 Å radius of the designated residues throughout the trajectory are shown for WT (A) and W75,86,160,221F (B) barrels. Lys-120 and Leu-270 served as controls for water-solvated and DDM-bound regions, respectively. The residue number is indicated within each panel. Comparing the total number of neighboring DDM (upper panels) and water molecules (lower panels) within a 5 Å radius of each tryptophan shows that 160^th^ position has high DDM content, comparable to that of the completely buried Leu-270 residue. This highlights the affinity of Trp-160 for a hydrophobic environment. At the same time, this position has a similar water content as the other tryptophans (75, 86, 221; see lower panels), reflecting the interfacial nature of this region. Similar results were obtained for WT and W75,86,160,221F simulations. Also see Supplementary Fig. 10 for more information. (C) Cartoon representation of hVDAC-2 WT barrel showing the positions of four intrinsic tryptophans (red sticks) and the control residues, K120 and L270 (purple sticks). (D) Cartoon representation of hVDAC-2 WT highlighting the neighboring aromatic residues (green sticks), and possible quenchers (yellow sticks), for each of the four tryptophans (red sticks). Possible quenchers for tryptophan fluorescence include residues like cysteine, histidine, glutamic acid and aspartic acid. The membrane position is shown as grey spheres.

Next, we examined the vicinity of each of the four indole (or the corresponding benzyl ring in the Trp-less barrel) positions for gauging the hydration levels and interaction with DDM molecules, over the course of the simulation. In general, we observe an inverse relationship between the hydration level and DDM molecules at the vicinity of both tryptophan (in WT) and phenylalanine (in the Trp-less construct) (Fig. 8A and B). A closer examination of the data reveals that although the 160^th^ position is at the interface, we observe a larger number of DDM molecules in its vicinity. The 160^th^ position is therefore comparable to the vicinity of a fully buried lipid-facing residue such as Leu-270, in its DDM content (Fig. 8A and B and Supplementary Fig. 10). Concomitantly, it is also well-hydrated, reflecting the amphipathic nature of this segment (Fig. 8A and B and Supplementary Fig. 10). Residue 160 lies at the start of β10. Both β9 and β10 are the most hydrophobic strands of the barrel, and are expected to bind DDM molecules (Fig. 6). An amphipathic residue at 160^th^ position may, therefore, be important to facilitate strand reversal at the β9–β10 junction and allow for solvation. However, water accessibility in this region also causes the destabilization of hVDAC-2 W75,86,221F upon thermal denaturation, particularly when the other anchoring indoles are absent.

The regions corresponding to 75 and 221 have almost similar number of DDM and water molecules (Fig. 8A and B and Supplementary Fig. 10), and could account for comparable stabilization by Trp or Phe in these positions. These tryptophans are also surrounded by residues like cysteines and glutamic acid that provide an amphipathic environment at the solvent-membrane interface (Fig. 8D). It is known that aromatic interaction networks at the interface sites in transmembrane β-barrels contribute considerably towards its stability [11]. We find that both Trp-75 and Trp-160 have three aromatic residues within a 5 Å distance, while Trp-221 has one (Fig. 8D). The formation of aromatic interactions is one likely mechanism by which the positions 75, 160 and 221 together stabilize the barrel. In contrast to Trp-75, the 86^th^ position only retains a considerable number of DDM molecules in its vicinity (Fig. 8A and B and Supplementary Fig. 10). Trp-86 has no aromatic residues in its vicinity, again supporting the destabilizing effect of this position.

## 4 Discussion

**Fig. 9:**
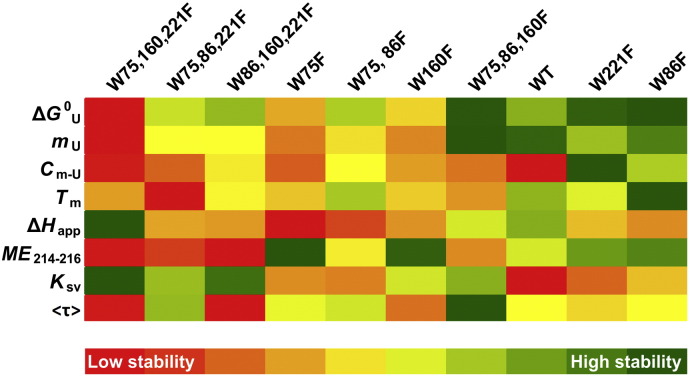
Stability profiles of hVDAC-2 WT and its tryptophan mutants. Parameters are listed to the left, and each mutant is color coded using a gradient of reds if they represent barrel destabilization, and a gradient of greens if they stabilize the folded conformation. The single tryptophan mutant W75,160,221F is the most unstable mutant (extreme left) while its counterpart W86F is highly stabilized (extreme right). Hence hVDAC-2 barrel stability increases if phenylalanine is placed at the 86^th^ position. W75,86,221F shows conditional destabilization in thermal denaturation measurements. Both tryptophan and phenylalanine stabilize the barrel when present at the 75^th^ and 221^st^ positions.

The contribution of ‘aromatic belts’ formed by tryptophans and tyrosines to membrane protein stability is considerable, and has been studied in detail [2], [3], [4]. Herein, we have tried to decipher the role of the four intrinsic conserved tryptophans of the human OMM protein VDAC-2, towards its stability in detergent micelles. From our biophysical analyses, it is evident that the contribution of these tryptophans is clearly dependent upon the position. While all the tryptophans are at the interface in hVDAC-2, our studies show that each position is unique and poses an intrinsic influence on the barrel stability. At the same time, it is also modulated by the placement of either tryptophan or phenylalanine at the other interface positions. This is summarized in Fig. 9, where the various biophysical parameters derived in this study are compared across the mutants.

The hVDAC-2 barrel structure is asymmetric and very similar to VDAC-1, with a height of ~ 4 nm, and a concave pore of dimensions ~ 3.5 nm × ~ 3.1 nm at the interface [28], [50], [51]. While all four indoles are at the interface, their contributions can be influenced by the local environment. The 86^th^ position tryptophan is buried as compared to the other three positions, and does not have any neighboring aromatic residues (Fig. 8D). The lone tryptophan at this position is considerably inefficient at supporting barrel folding (Fig. 3) and along with W86,160,221F, is the mutant with the least secondary structure content (Fig. 3C). Additionally, this mutant has the least chemical stability (Fig. 4). When the more hydrophobic phenylalanine is placed at this position the barrel stability increases manifold (Fig. 9) owing to its better adjustment to the hydrophobic lipidic interior. Hence, we find that in the presence of Trp-86, and absence of other three intrinsic tryptophans, the β4 strand is destabilized. However, Trp-86 shows absolute conservation in most of the known VDAC sequences [52], indicating a possible functional role for β4 destabilization that is currently unknown.

A tryptophan at residue 75 is seen in VDAC-1 and -2, but is replaced by aliphatic residues in isoform 3 [52]. hVDAC-2 barrel stability is moderately affected when substituted with phenylalanine (see the adjacent placement of W86,160,221F and W75F in Fig. 9). However, Trp-75 does cause a marginal destabilization of β3. Similarly, both Phe and Trp are tolerated at position 221. However, both Trp-160 and Trp-221 are highly conserved across all three VDAC isoforms from various organisms [52]. The importance of these regions is also highlighted by its functional relevance in Bak import, tBid induced apoptosis and interaction with steroidogenic acute regulatory protein [53], [54]. The 221^st^ position is close to one of the longest loop of the hVDAC-2 barrel. Along with Trp-160, Trp-221 can drive folding of the hVDAC-2 barrel (Fig. 3). While the W221F mutant shows an unusual increase in the barrel’s resistance to chemical solvation (see Fig. 4), we believe that this is due to the intrinsic hydrophobic nature of phenylalanine. Nevertheless, the 221^st^ position can accommodate either tryptophan or phenylalanine in the folded protein, as observed from the close placement of W75,86,160F and W221F in the color scale (Fig. 9).

The true amphipathic nature of tryptophan plays an important role at the 160^th^ position. Although this position is at the interface, it has a hydrophobic surrounding. The nearby aromatic residues provide additional stability to this part of the barrel by forming aromatic interactions (Fig. 8D). The presence of tryptophan only at this position compromises the barrel thermal stability (Fig. 5). We believe that this phenomenon is due to water molecules gaining easier access into the barrel-micelle junctions upon application of heat, when the amphipathic tryptophan is present. The apparent overall stability of the barrel is increased when phenylalanine is present at this position (Fig. 9), as it is able to better adjust itself in the hydrophobic neighborhood.

Overall, it seems that replacement of tryptophan with a phenylalanine causes no change or increases the overall stability of hVDAC-2 barrel. However, the Trp-less mutant shows decreased folding efficiency and secondary structure content. Trp → Phe mutation also introduces hysteresis in the hVDAC-2 barrel that is otherwise maintained under thermodynamic equilibrium. Evolution has thus retained tryptophans at these positions to form a functional and stable barrel in the OMM that is also structurally malleable. It has also not escaped our notice that our study has provided the first insight into the *in vitro* hVDAC-2 folding mechanism. Our experiments show that the C-terminal segments, namely strands β14 and β10, are likely to fold first. This is also supported by the hydrophobic nature of the strands β9–10 and β15–18 (see Fig. 6), which allows this region of the protein to form strong protein-lipid association. The nucleation of folding at the C-terminal strands can then propagate towards the N-terminal strand segments. The slow folding rate of mutant with only Trp-86 also supports our conjecture.

Mitochondria are subjected to considerable chemical (oxidative) stress in the cellular environment, owing to its close association with the metabolic redox machinery [55]. These insults, in turn, can lead to protein oxidation or alteration in protein behavior that can impact overall cellular homeostasis [17]. The formation of a more dynamic VDAC-2 barrel, facilitated by the interface tryptophans is likely to be chosen during evolution to help the mitochondrion in maintaining VDAC levels in the OMM and in rapidly regulating protein turnover.

## Funding

This work is supported by the Wellcome Trust/ DBT India Alliance award number IA/I/14/1/501305 to R.M.

## Transparency document

Transparency document. Image 2

## Figures and Tables

**Table 1:** hVDAC-2 and its tryptophan mutants.

hVDAC-2	Residue and position				hVDAC-2 mutant description
	75	86	160	221	
WT	**W**	**W**	**W**	**W**	Wild-type hVDAC-2; intrinsic tryptophans at positions 75, 86, 160, 221
W86,160,221F	**W**	F	F	F	hVDAC-2 W86F, W160F, W221F
W75,160,221F	F	**W**	F	F	hVDAC-2 W75F, W160F, W221F
W75,86,221F	F	F	**W**	F	hVDAC-2 W75F, W86F, W221F
W75,86,160F	F	F	F	**W**	hVDAC-2 W75F, W86F, W160F
W75,86F	F	F	**W**	**W**	hVDAC-2 W75F, W86F
W75F	F	**W**	**W**	**W**	hVDAC-2 W75F
W86F	**W**	F	**W**	**W**	hVDAC-2 W86F
W160F	**W**	**W**	F	**W**	hVDAC-2 W160F
W221F	**W**	**W**	**W**	F	hVDAC-2 W221F
W75,86,160,221F	F	F	F	F	hVDAC-2 W75F, W86F, W160F, W221F

**Table 2:** Voltage gating parameters for hVDAC-2 WT and its single tryptophan mutants.

hVDAC-2 mutants^a^	Positive voltages			Negative voltages			Single channel conductance [nS]^d^
	*n*	*V*_0_^b^	*nFV*_0_^c^	*n*	*V*_0_^b^	*nFV*_0_^c^	
WT (5)^e^	3.12 ± 0.50	28.63 ± 1.92	8.62	3.07 ± 0.47	−25.00 ± 2.64	7.4	2.43 ± 0.58 (6), 3.98 ± 0.50 (12)
W86,160,221F (4)	2.54 ± 0.55	19.97 ± 1.00	4.90	1.89 ± 0.33	−22.13 ± 2.90	4.04	2.44 ± 0.43 (5), 3.97 ± 0.63 (10)
W75,160,221F (4)	2.80 ± 0.71	24.85 ± 3.37	6.72	3.13 ± 0.31	−22.59 ± 2.93	6.82	2.48 ± 0.26 (6), 3.85 ± 0.39 (10)
W75,86,221F (4)	2.58 ± 0.20	23.64 ± 2.85	5.89	2.31 ± 0.20	−26.07 ± 1.86	5.80	2.82 ± 0.21 (6), 3.91 ± 0.37 (8)
W75,86,160F (4)	2.24 ± 0.61	25.63 ± 2.44	5.55	2.29 ± 0.25	−24.08 ± 3.22	5.33	2.46 ± 0.57 (5), 3.74 ± 0.47 (11)

Error values represent standard deviation between independent experiments.

a Values in brackets indicate the number of independent experiments conducted for the voltage ramp studies to derive n and *V*_0_ values.

b *V*_0_ given in mV.

c *nFV*_0_ given in kJ mol^−1^.

d Numbers in parenthesis indicate the total number of channels considered from at least three independent experiments. Channels are segregated based on their insertion in fully open (~ 4 nS) or in subconductance (~ 2 nS) states.

e Parameters for WT are published in reference [19] and are used here with permission for purpose of comparison.

## Appendix A Supplementary data

Supplementary material Image 3
