# Supplementary material for: Control of human VDAC-2 scaffold dynamics by interfacial tryptophans is position specific
Source: Biochim Biophys Acta. 2016 Dec;1858(12):2993–3004. doi: 10.1016/j.bbamem.2016.09.011 (PMC5091009; doi:10.1016/j.bbamem.2016.09.011)
Supplement: Supplementary file 2 — Supplementary material [file mmc2.pdf]

Control of human VDAC-2 scaffold dynamics by interfacial tryptophans is position specific

Svetlana Rajkumar Maurya and Radhakrishnan Mahalakshmi*

Molecular Biophysics Laboratory, Department of Biological Sciences, Indian Institute of Science Education and Research, Bhopal. India. *E-mail: maha@iiserb.ac.in

SUPPLEMENTARY INFORMATION

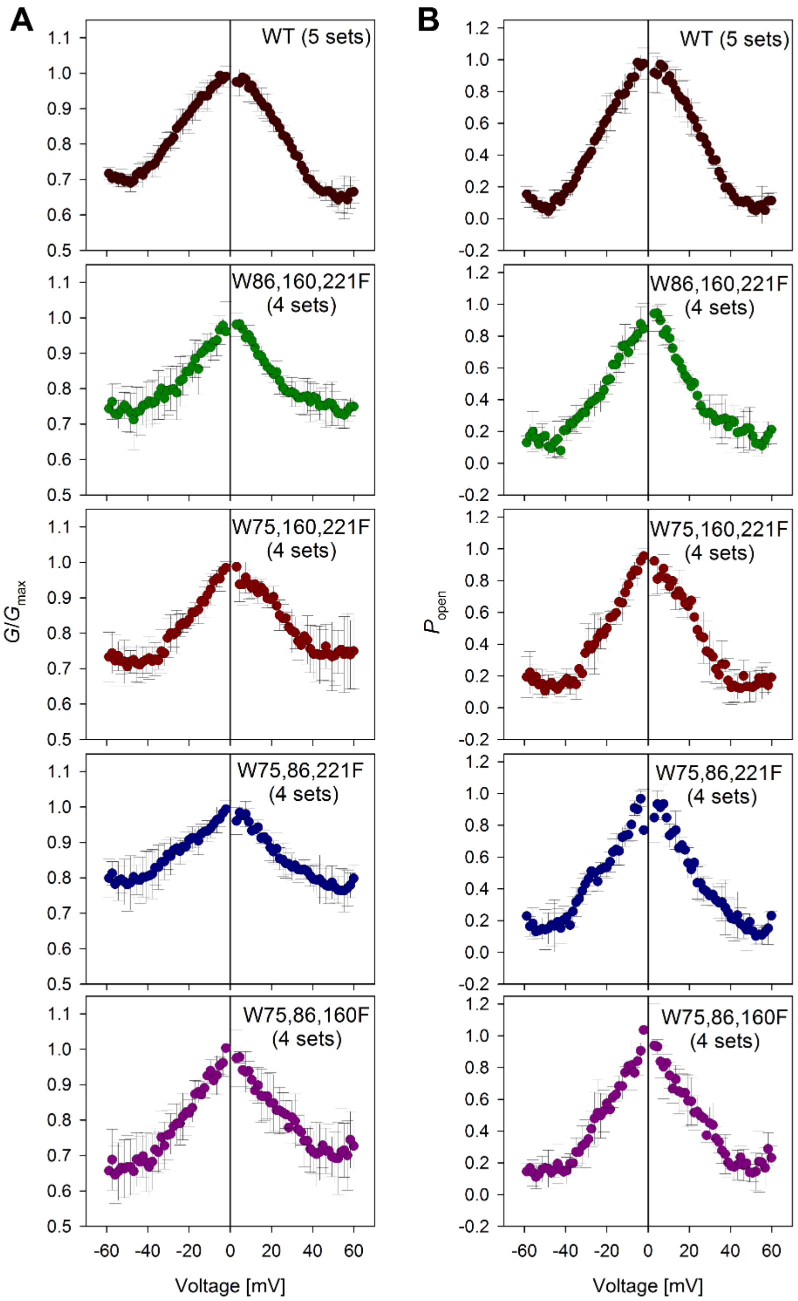

Supplementary Fig. 1. Planar bilayer measurements of hVDAC-2 WT and its single tryptophan mutants showing (A) G/G_{max} and (B) open probability (P_{open}) measurements derived from multi-channel membranes. The subconductance state of W75,86,221F (having Trp-160) is different from the other Trp mutants, as the barrel exhibits the poorest response to higher voltages (left panels). The open probability remains comparable for all mutants. Numbers in brackets indicate the total number of independent experiments considered and error bars indicate standard deviations from those experiments. Data for hVDAC-2 WT protein has been reproduced from reference [1] with permission.

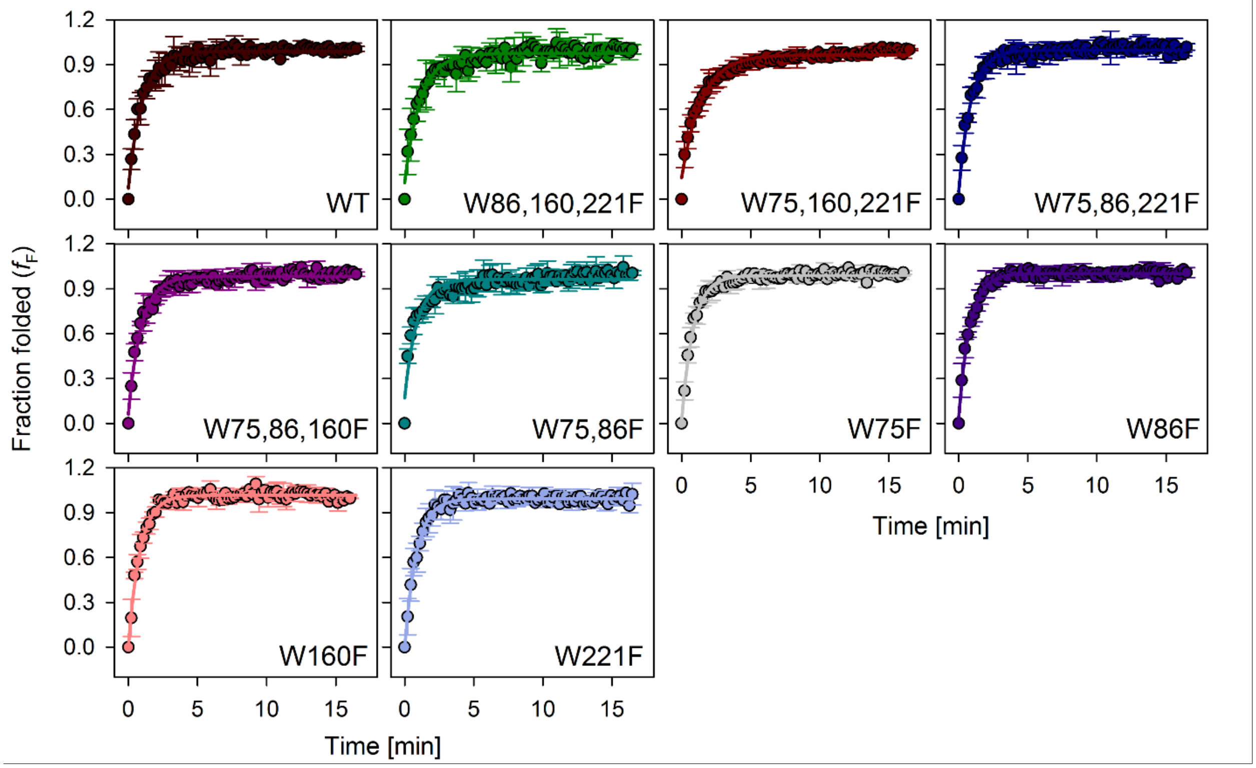

Supplementary Fig. 2. Folding kinetics of hVDAC-2 WT and tryptophan mutants in 19.5 mM DDM at 4 °C, monitored using Trp fluorescence anisotropy. Anisotropy measurements were converted to folded fraction as described in the Materials and Methods section. Data for the single tryptophan mutants could also be fitted to a double exponential function. However, consistency among independent datasets was not observed. Hence, only single exponential function was used for fitting (represented as solid lines) for purposes of comparison across all mutants. Error bars denote standard deviation from three independent data sets.

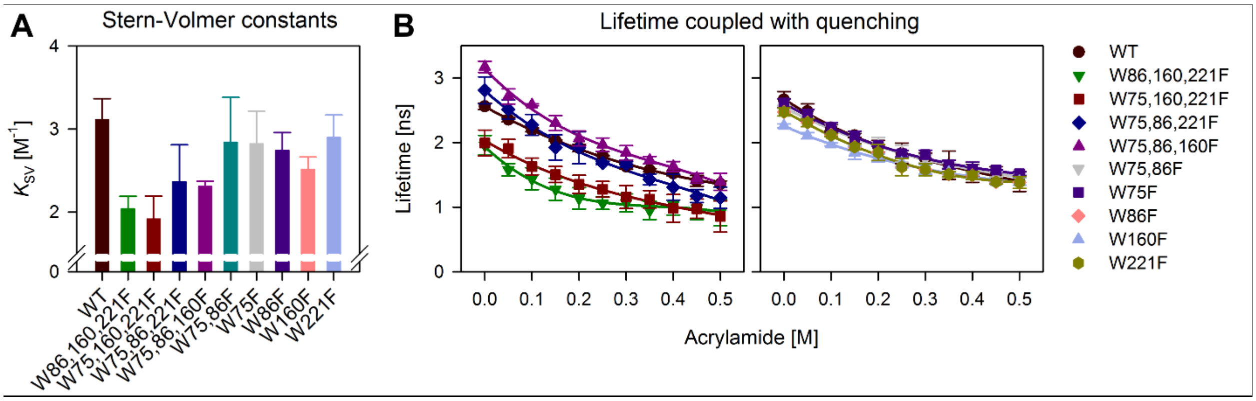

Supplementary Fig. 3. Acrylamide quenching coupled with tryptophan fluorescence and lifetime of folded hVDAC-2 WT and mutant barrels in DDM micelles. (A) Stern-Volmer constants (K_{SV}) obtained from steady-state fluorescence data shows that all single tryptophan mutants, especially W86,160,221F and W75,160,221F, have very low K_{SV} values. Lowered K_{SV} indicates a buried nature of the tryptophan. (B) Tryptophan lifetime coupled with acrylamide quenching for all the single tryptophan (left panel) and multi-tryptophan (right panel) proteins. hVDAC-2 WT values are shown in both the panels for purpose of comparison. The lifetime of W86,160,221F shows a rapid decrease and reaches saturation at acrylamide concentrations of ~0.3 M, indicating the likely presence of a local hydrophilic residue in its vicinity, which may act as an additional quencher for Trp fluorescence.

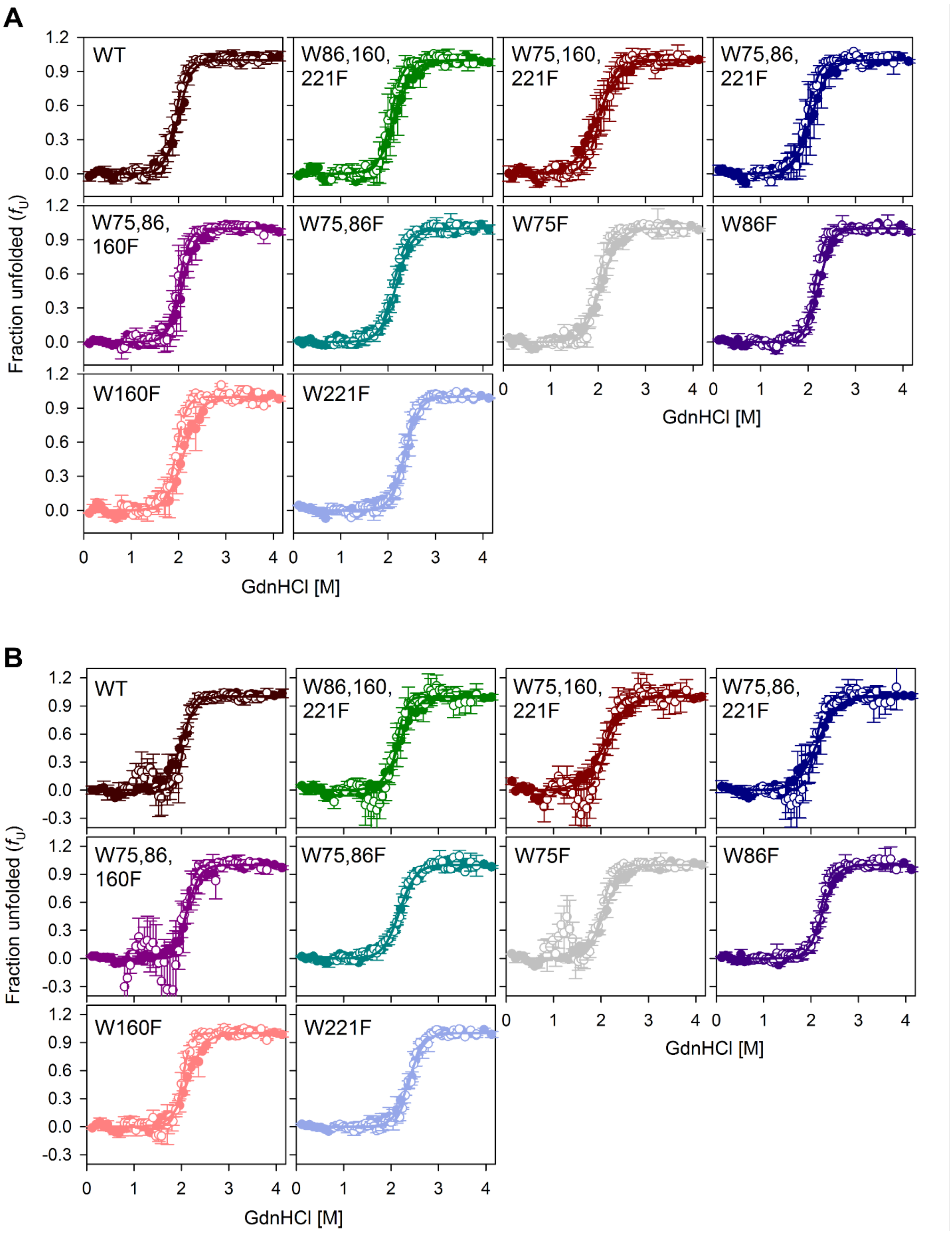

Supplementary Fig. 4. Equilibrium (un)folding experiments of hVDAC-2 WT and its tryptophan mutants using GdnHCl, at 24 h (A) and 72 h (B) in 3.9 mM DDM at 25 °C. (A)

While WT barrel does not show prominent hysteresis at 24 h, some of the mutants, especially W160F, shows considerable hysteresis. The absence of selected tryptophan residues, such as Trp-160, can offset the thermodynamic equilibrium of the barrel by either affecting the unfolding or folding pathways. (B) When we increase the incubation time, we see considerable protein aggregation at intermediate GdnHCl concentrations (1-2 M) that mainly affects the folding reaction. The fluorescence intensity profiles at 72 h are shown here for purpose of representation. The folding profiles of the mutants W75,86F, W86F, W160F and W221F do not get affected, although we can visualize the aggregation at similar GdnHCl concentrations. Filled and empty circles represent unfolding and folding reactions, respectively. Solid and dashed lines indicate fits to the two-state equation for the unfolding and folding reactions, respectively. Error bars denote standard deviation from at least two independent experiments.

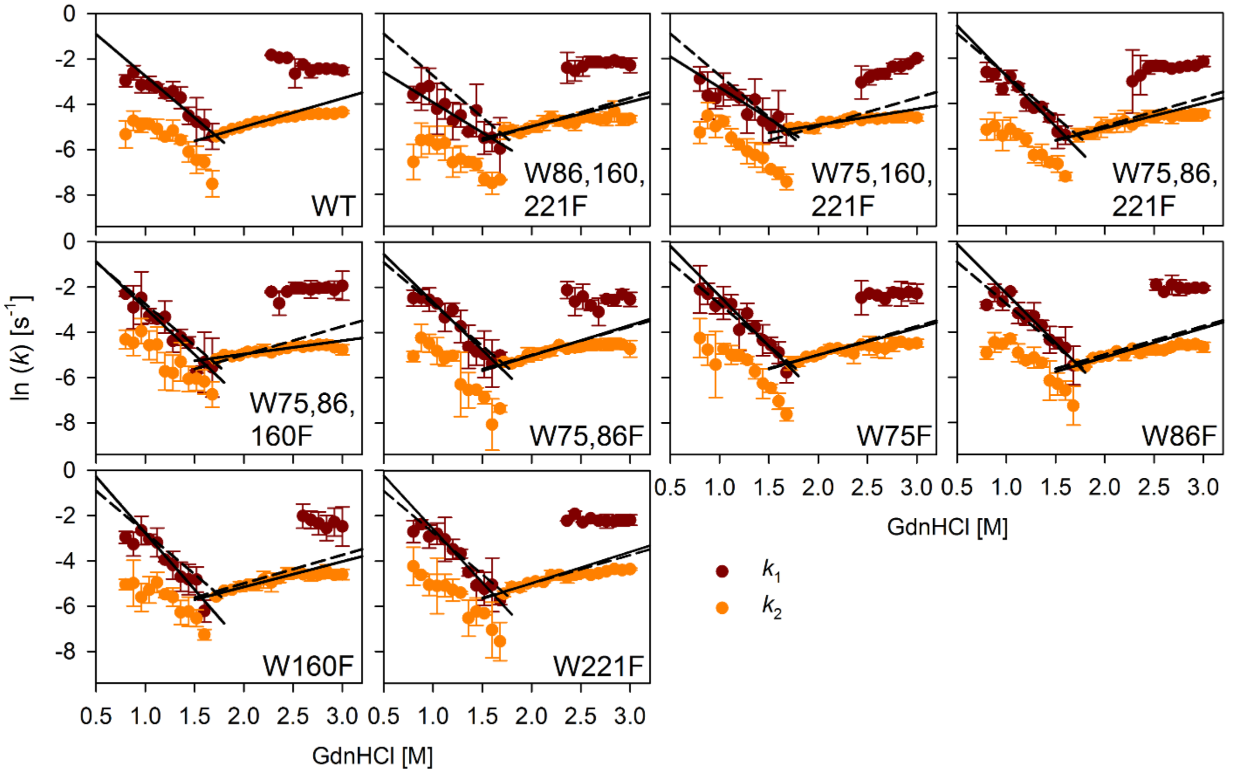

Supplementary Fig. 5. Chevron plots for hVDAC-2 WT and tryptophan mutants in 3.9 mM DDM at 25 °C. The folding arm was generated by fitting the folding kinetics to a double exponential function. Unfolding kinetics from 1.68 M to ~2.3 M fit well to a single exponential function and at higher GdnHCl concentrations, double exponential function was used for fitting. Both the arms showed rollovers, with the unfolding arm rollover being more prominent. Only the linear zone was fit to a two state Chevron function (solid lines) to derive the thermodynamic parameters summarized in Supplementary Table 1. The fits for the hVDAC-2 WT protein are shown as dotted lines in all the mutant datasets, for comparison.

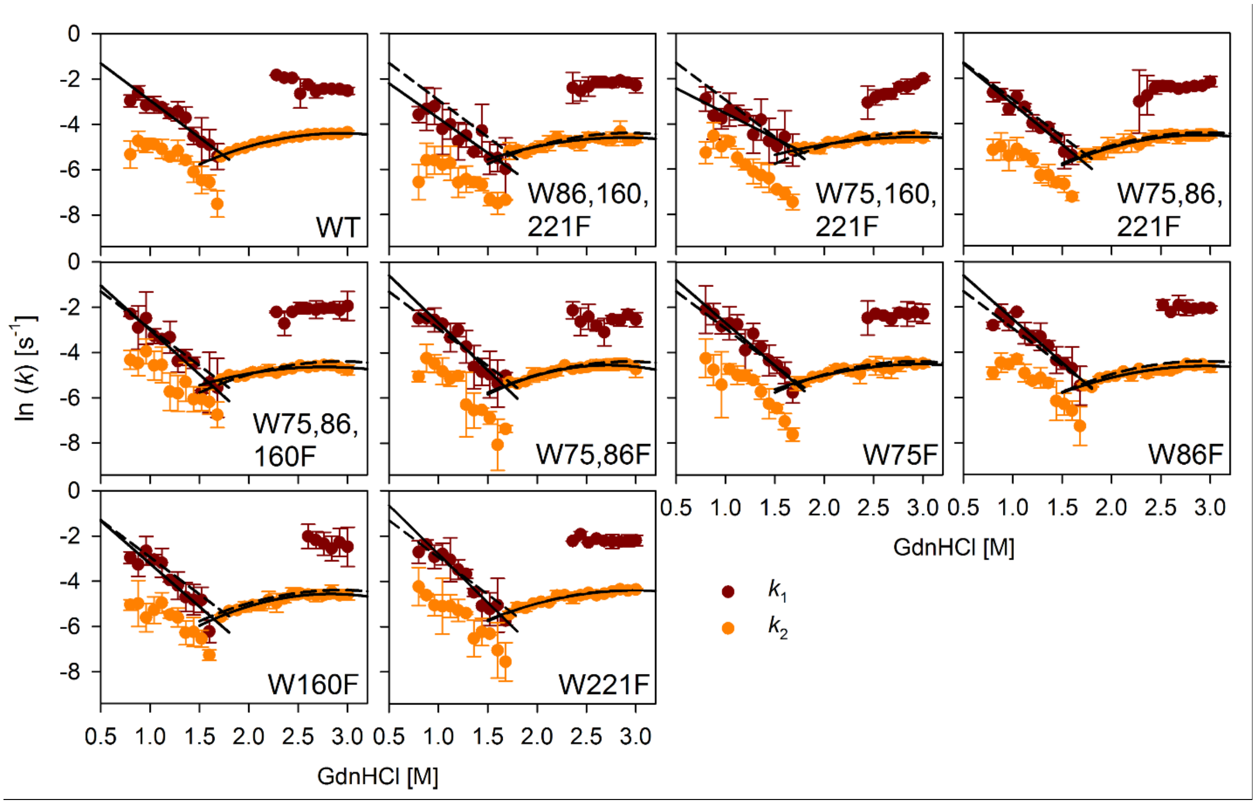

Supplementary Fig. 6. Chevron plots for hVDAC-2 WT and tryptophan mutants in 3.9 mM DDM at 25 °C. The unfolding arm in this case has been fit to a rollover function (solid lines) to account for the kink in the unfolding arm. The marginal rollover in the folding arm is not considered here, as it is likely to arise due to protein aggregation at lower GdnHCl concentrations [1]. The values for thermodynamic parameters are summarized in Supplementary Table 1. The fits for the hVDAC-2 WT protein are shown as dotted lines in all the mutant datasets, for comparison.

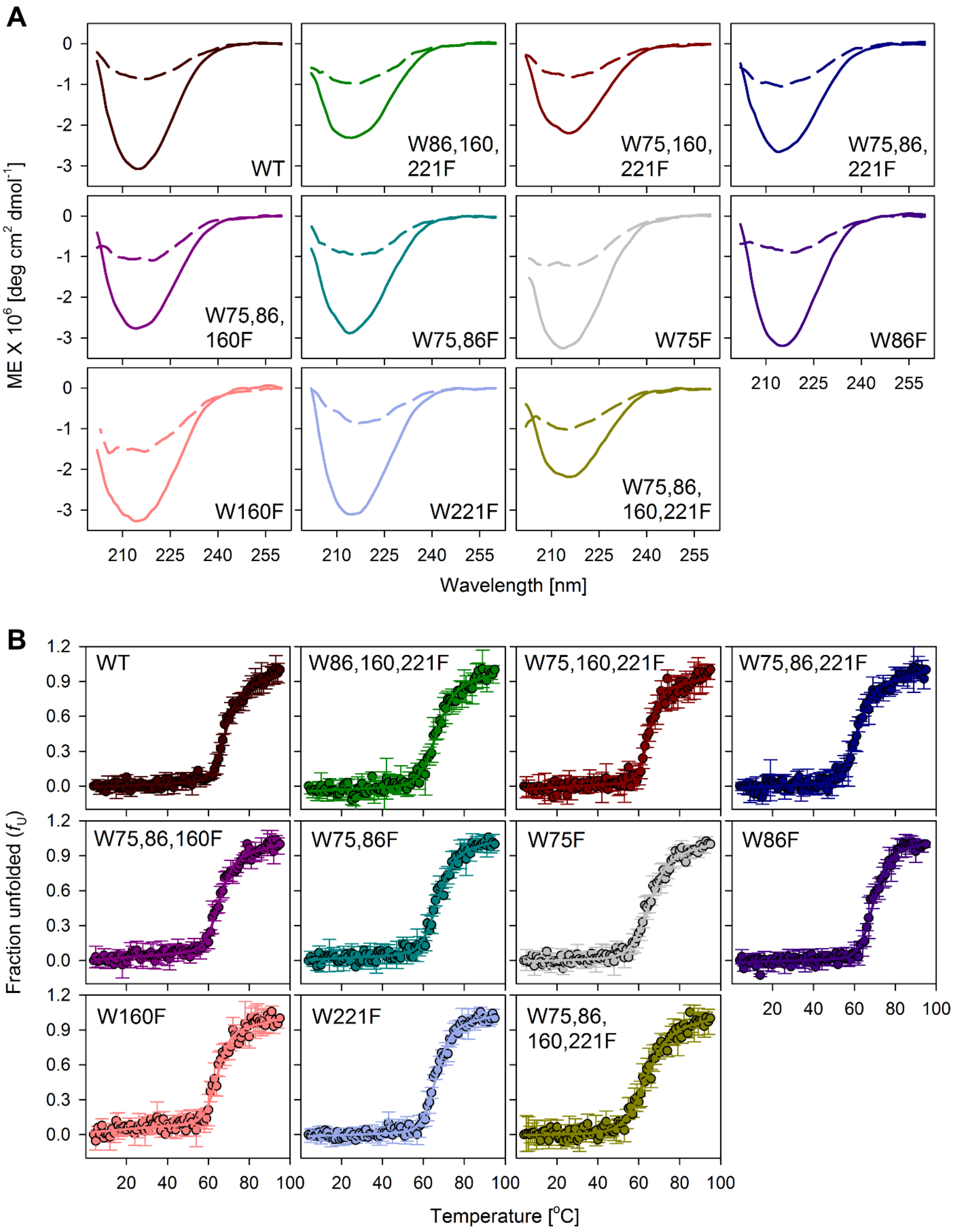

Supplementary Fig. 7. Representative far-UV CD wavelength scans (A) and thermal denaturation profiles (B) of folded hVDAC-2 WT and its tryptophan mutants in 3.9 mM DDM.

(A) Considerable loss in secondary structure content is observed when folded hVDAC-2 is heated till 95 °C, as seen from the before (solid line) and after melting (dashed line) wavelength scans. (B) Data were acquired from 4-95 °C, monitored at 215 nm. Unfolded fractions at each temperature were calculated using reported methods [1]. Solid lines represent fits of the unfolded fractions to a two-state function. Error bars in (B) indicate standard deviation obtained from at least two independent experiments.

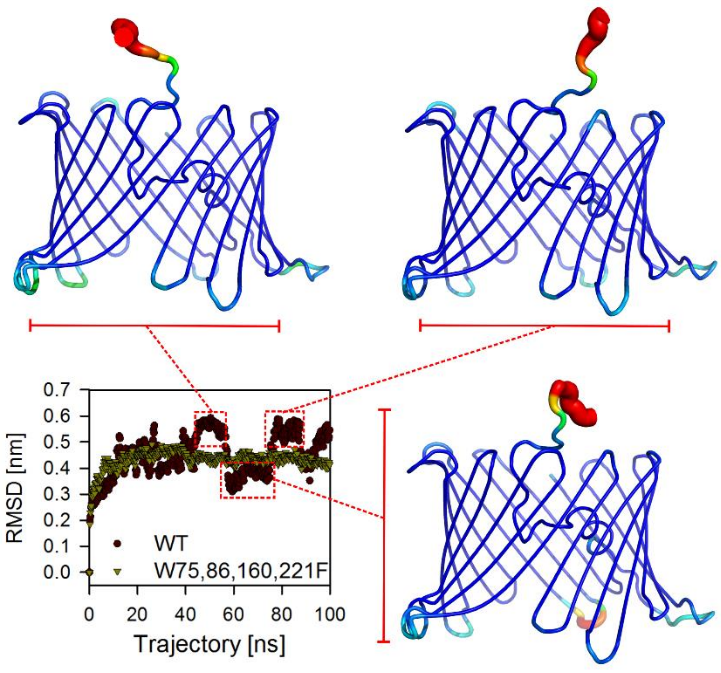

Supplementary Fig. 8. *In silico* analysis of root mean square deviation (RMSD) of hVDAC-2 WT barrel. Whole protein RMSD of hVDAC-2 WT shows more fluctuations as compared to the Trp-less protein (lower left panel, part of Fig. 7A) due to dynamic nature of the N-terminal 11 residues. The RMSD plot of WT shows three stable zones that are highlighted in red dotted boxes. The corresponding sausage models derived from each of these zones highlight the differences in the orientation of the N-terminal portion of the WT protein, while the topology of rest of the barrel remains largely unchanged.

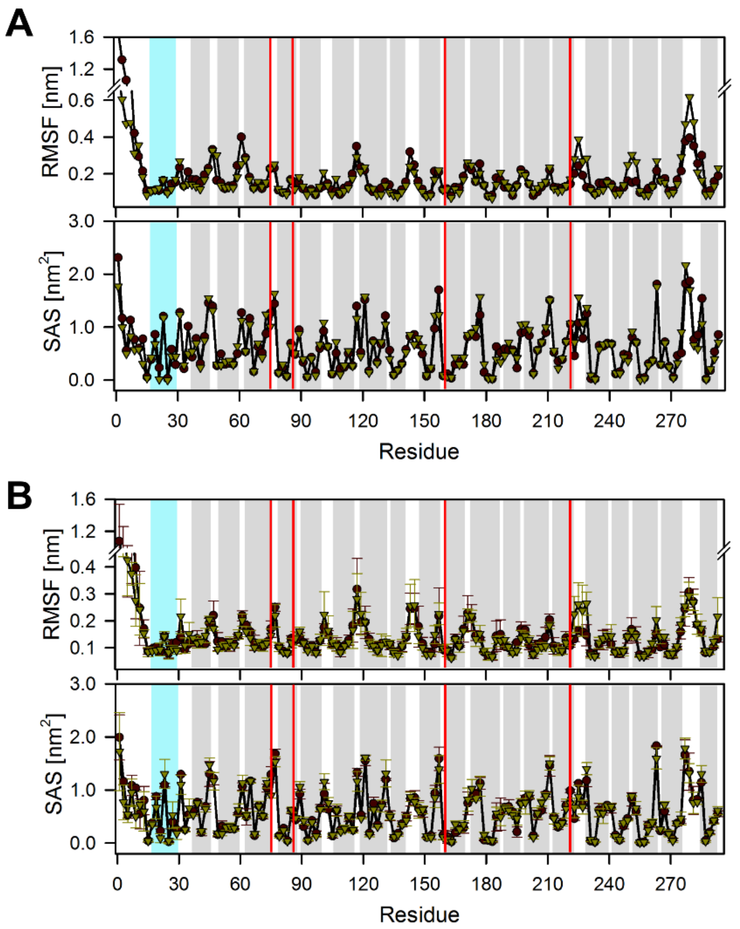

Supplementary Fig. 9. Root mean square fluctuation (RMSF) and solvent accessible surface (SAS) area calculated from MD simulations of 100 ns (A) and 10 ns (B) for hVDAC-2 WT (brown circles) and W75,86,160,221F (Trp-less mutant, olive triangles). Higher values of RMSF and SAS are localized to residues present in loop regions in both the 10 ns and 100 ns simulations. The grey bars indicate the β-strands, white spaces correspond to the loop regions, and red lines indicate the positions 75, 86, 160 and 221. The N-terminal helix is found between residues ~17-29, and is indicated in cyan. Error bars in (B) indicate standard deviation and are derived from three independent 10 ns simulations.

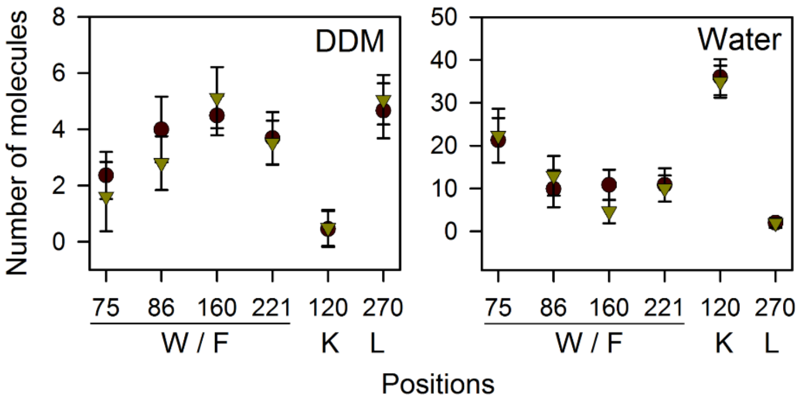

Supplementary Fig. 10. Vicinity analysis of Trp/Phe at positions 75, 86, 160 and 221 in hVDAC-2 WT (brown circles) and W75,86,160,221F (Trp-less mutant, olive triangles) obtained from a 100 ns simulation in DDM micelle. Lys-120 and Leu-270 serve as controls for water-solvated and DDM-bound residues, respectively. The 86th and 160th positions attract a high number of DDM molecules (left panel), which is comparable to the buried Leu-270 residue in both WT and W75,86,160,221F barrels. The 75th position shows marginally increased water content (right panel) in its vicinity when compared to other Trp/Phe positions. Error bars denote standard deviation obtained from averaging the values of 1000 frames throughout the 100 ns trajectory.

Supplementary Table 1: Thermodynamic parameters obtained from chevron plots.

Parameters obtained from linear fits of both arms[@]									
**hVDAC-2 mutants**	**ln(k_F)**	**m_F**	**ln(k_U)**	**m_U**	**m_{U1}**	**ΔG^0_{kin}**	**m**	**C_m**	**β_T**
**WT**	0.94	-2.18	-7.51	0.74	-	-5.00	2.93	1.71	0.75
**W86,160,221F**	-1.27	-1.57	-7.15	0.64	-	-3.48	2.22	1.57	0.71
**W75,160,221F**	-0.55	-1.62	-6.33	0.41	-	-3.42	2.03	1.69	0.80
**W75,86,221F**	1.67	-2.64	-7.29	0.65	-	-5.31	3.29	1.61	0.80
**W75,86,160F**	1.19	-2.44	-6.14	0.35	-	-4.34	2.79	1.56	0.87
**W75,86F**	1.57	-2.50	-7.70	0.79	-	-5.49	3.30	1.66	0.76
**W75F**	2.03	-2.62	-7.38	0.70	-	-5.57	3.32	1.68	0.79
**W86F**	2.08	-2.59	-7.69	0.76	-	-5.78	3.35	1.73	0.77
**W160F**	2.22	-2.96	-7.40	0.67	-	-5.70	3.62	1.57	0.82
**W221F**	2.14	-2.80	-7.74	0.82	-	-5.85	3.62	1.62	0.77
**Parameters obtained from rollover equation in unfolding arm[@,#]**									
**hVDAC-2 mutants**	**ln(k_F)**	**m_F**	**ln(k_U)**	**m_U**	**m_{U1}**	**ΔG^0_{kin}**	**m**	**C_m**	**β_T**
**WT**	0.94	-2.18	-10.16	2.33	-0.40	-6.57	4.51	1.46	0.48
**W86,160,221F**	-1.27	-1.57	-8.82	1.74	-0.30	-4.47	3.31	1.35	0.48
**W75,160,221F**	-0.55	-1.62	-7.87	1.32	-0.23	-4.33	2.94	1.47	0.55
**W75,86,221F**	1.67	-2.64	-10.22	2.33	-0.40	-7.04	4.97	1.42	0.53
**W75,86,160F**	1.19	-2.44	-8.52	1.66	-0.30	-5.75	4.10	1.40	0.59
**W75,86F**	1.57	-2.50	-10.98	2.80	-0.51	-7.43	5.30	1.40	0.47
**W75F**	2.03	-2.62	-9.03	1.79	-0.30	-6.54	4.41	1.48	0.59
**W86F**	2.08	-2.59	-9.44	1.93	-0.33	-6.82	4.52	1.51	0.57
**W160F**	2.22	-2.96	-10.90	2.65	-0.47	-7.77	5.61	1.39	0.53
**W221F**	2.14	-2.80	-9.54	2.02	-0.34	-6.92	4.82	1.44	0.58
Units: k_F, k_U: min^{-1}; m_F, m_U, m_{U1}, m: kcal/mol/M; ΔG^0_{kin}: kcal/mol; C_m: M.<br>[@]*m* values presented here are from the kinetic experiments.<br>[#]The trend obtained for ΔG^0_{kin} using the rollover equation is similar to the equilibrium data.									

References

[1] S.R. Maurya, R. Mahalakshmi, N-helix and Cysteines Inter-regulate Human Mitochondrial VDAC-2 Function and Biochemistry, J. Biol. Chem. 290 (2015) 30240-30252.
